# ﻿The subfamily Chalciporoideae (Boletaceae, Boletales) in China

**DOI:** 10.3897/mycokeys.124.165901

**Published:** 2025-10-22

**Authors:** Xu Zhang, Xin-Ni Li, Wei-Qing Liang, Xiao-Dong Mu, Ye-Fei Yu, Xiao-Jun Wu, Si-Yu Chen, Jin-Bao Pu, Nian-Kai Zeng

**Affiliations:** 1 Ministry of Education Key Laboratory for Ecology of Tropical Islands, Key Laboratory of Tropical Animal and Plant Ecology of Hainan Province, College of Life Sciences, Hainan Normal University, Haikou, China Hainan Normal University Haikou China; 2 Center for Medicinal Resources Research, Zhejiang Academy of Traditional Chinese Medicine, Hangzhou, China Center for Medicinal Resources Research, Zhejiang Academy of Traditional Chinese Medicine Hangzhou China; 3 Zhejiang Engineering Research Center for Quality Assessment and Development of Dao-di Herbs, Hangzhou, China Zhejiang Engineering Research Center for Quality Assessment and Development of Dao-di Herbs Hangzhou China; 4 Hainan Research Academy of Environmental Sciences, Haikou, China Hainan Research Academy of Environmental Sciences Haikou China; 5 Zhejiang Dapanshan National Natural Reserve Administration, Panan, China Zhejiang Dapanshan National Natural Reserve Administration Panan China

**Keywords:** Bolete, molecular phylogeny, morphology, new taxa, taxonomy

## Abstract

The subfamily Chalciporoideae, an early diverging lineage within Boletaceae (Boletales), is ecologically significant and economically promising. However, research on species diversity within the subfamily in China is still insufficient. In this study, detailed morphological examinations and molecular phylogenetic analyses were conducted on specimens collected from various regions in China. Three genera in Chalciporoideae, viz. *Buchwaldoboletus*, *Chalciporus*, and *Pseudophylloporus* were confirmed to be distributed in China; 16 species of the subfamily were confirmed to occur in China. Among the species recognized in China, four were newly described, viz. *Chalciporus aurantiolepidotus*, *C. brunneus*, *C. roseus*, and *Pseudophylloporus castaneus*. Two known species were redescribed, and eight other species were reviewed. Keys to the accepted species of *Buchwaldoboletus*, *Chalciporus*, and *Pseudophylloporus* were also provided, respectively.

## ﻿Introduction

The subfamily Chalciporoideae represents one of the earliest diverging lineages within Boletaceae (Boletales). It currently includes the genera *Chalciporus* Bataille, *Buchwaldoboletus* Pilát, and the recently described *Pseudophylloporus* N.K. Zeng, H.Z. Qin, W.F. Lin & L.G. Hu (Nuhn et al. 2013; Wu et al. 2016; Qin et al. 2024; Tremble et al. 2024). Species within this subfamily are widely distributed worldwide, with particularly high diversity in tropical and subtropical regions (Desjardin et al. 2009; Nuhn et al. 2013; Raspé et al. 2016; Wu et al. 2016; Zhang et al. 2016, 2017; Xie et al. 2021; Qin et al. 2024). However, unlike most Boletaceae members that form ectomycorrhizal symbioses, the trophic modes of Chalciporoideae species are complex and not yet fully understood. Studies suggest that species within this subfamily may adopt various nutritional strategies, including ectomycorrhizal, saprotrophic, or mycoparasitic modes (Degreef and De Kesel 2008; Dickie et al. 2010, 2016; Migliorini et al. 2012). For example, *C. piperatus* (Bull.) Bataille was historically regarded as a typical ectomycorrhizal fungus, but recent ecological observations, isotopic analyses, and artificial synthesis experiments indicate that it is more likely a mycoparasitic or saprotrophic fungus (Dickie and Johnston 2008; Dickie et al. 2010; Robinson 2010). Similarly, *B. lignicola* (Kallenb.) Pilát, the type species of *Buchwaldoboletus*, has been shown to function both as a mycoparasite that attacks wood-decay fungi and as a saprotroph that directly decomposes wood, exhibiting a dual trophic mode of parasitism and saprotrophy (Caiafa and Smith 2022).

The species within Chalciporoideae also exhibit significant economic and edible potential. *Chalciporus piperatus* has been widely used as a food seasoning and in dye production (Li and Song 2002; Antonio 2003; Roberts and Evans 2011). *Buchwaldoboletus xylophilus* (Petch) Both & B. Ortiz has been successfully cultivated in artificial mushroom farms, becoming the second species within the Boletales worldwide to be cultivated artificially (Yang et al. 2025). It demonstrates outstanding performance in mycelial growth rate, yield, contaminant resistance, and biological conversion efficiency, highlighting its great potential for industrialization as an emerging edible mushroom resource (Yang et al. 2025).

In recent years, multiple new species of Chalciporoideae have been described worldwide, such as *C. perezsilvae* Pérez-Moreno, Ayala-Vásquez, Mart.-Reyes & C.R. Martínez-González, *C. piedracanteadensis* Ayala-Vásquez, Pérez-Moreno & Mart.-Reyes, *C. pseudopiperatus* Klofac & Krisai, and *C. rubrostipitatus* Nanu & T.K.A. Kumar (Klofac and Krisai-Greilhuber 2020; Ayala-Vásquez et al. 2023; Nanu and Kumar 2024). Chinese researchers have played a prominent role in these efforts, contributing to the description of several new taxa, including *C. sinensis* N.K. Zeng, Chang Xu, S. Jiang & Zhi Q. Liang, *C. vulparius* N.K. Zeng, Chang Xu & Zhi Q. Liang, and *P. baishanzuensis* N.K. Zeng, H.Z. Qin, W.F. Lin & L.G. Hu (Xu et al. 2021; Qin et al. 2024). Beyond these, investigations within China have revealed additional novel taxa in this subfamily. Prior to the current study, ten species of Chalciporoideae had been recorded from China (Wu et al. 2016; Zhang et al. 2016, 2017; Xie et al. 2021; Xu et al. 2021; Mao et al. 2023; Qin et al. 2024), significantly enhancing the known diversity of Chalciporoideae. Despite these advances, the diversity of the subfamily, taxonomic relationships, and phylogenetic position of many species remain unresolved. In this study, we conducted extensive species surveys across various regions of China. Through comprehensive morphological observations and multilocus phylogenetic analyses, we clarified the species composition and phylogenetic relationships of Chalciporoideae in China. The results not only led to the discovery of new taxa, but also included the redescription of two known species and a review of seven additional species, providing a solid foundation for further investigations into the diversity and taxonomy of this subfamily.

## ﻿Materials and methods

### ﻿Morphological studies

Digital photographs and field observations were recorded on fresh basidiomata during fieldwork. Collected specimens were dried at 50–60 °C and subsequently deposited in the Hainan Biodiversity Science and Technology Museum (**FHMU**), located in Haikou City, Hainan Province, China or the Herbarium of Medicinal Resources, Zhejiang Academy of Traditional Chinese Medicine (**ZJMR**), located in Hangzhou City, Zhejiang Province. Color descriptions follow the standard codes established by Kornerup and Wanscher (1981). Basidiomata were manually sectioned and examined microscopically after mounting in a 5% KOH solution using an Olympus CX23 optical microscope (Olympus, Tokyo, Japan). The annotation format [n/m/p] indicates the number of basidiospores (n) measured from m basidiomata belonging to p separate collections. Basidiospore measurements are reported as (a–)b–c(–d), where b–c represents the 5^th^ to 95^th^ percentile range of observed values, while a and d are exceptional values outside this range. The ratio of length to width is expressed as Q, with Qm representing the average Q value and its standard deviation. Descriptive terms for the size of basidiomata follow the conventions established by Bas (1969).

### ﻿Molecular procedures

For DNA extraction, small portions from the fresh basidiomata were cut, wrapped individually in paper, and sealed in bags containing silica gel to maintain dryness. Total genomic DNA was extracted from approximately 10–20 mg of dried fungal material using the Magnetic Beads Genomic DNA Extraction Kit (Magen, Guangzhou, China) according to the manufacturer’s instructions. Extracted DNA samples (2 µL each) were evaluated for concentration and purity using a NanoDrop 8000 spectrophotometer (Thermo Fisher Scientific, Waltham, MA, USA). To confirm DNA integrity, another aliquot (2 µL) was combined with an equal volume of bromophenol blue loading dye and electrophoresed on a 1% agarose gel in TAE buffer at 100 V for 20 minutes. Negative controls without DNA template were included in all extraction batches to detect potential contamination.

PCR amplification was performed on fragments of the nuclear ribosomal internal transcribed spacer (ITS), the large subunit ribosomal DNA (28S), the translation elongation factor 1-α (*TEF*1) and the RNA polymerase II second largest subunit gene (*RPB*2), employing primer pairs ITS5/ITS4 (White et al. 1990), LR0R/LR5 (Vilgalys and Hester 1990; James et al. 2006), tefF/tefR (Morehouse et al. 2003), and bRPB2-6F/bRPB2-7.1R (Matheny 2005), respectively. PCR reaction conditions were adapted from the protocols described by An et al. (2017) and Zhang et al. (2021). The 30 µL reaction mixture contained 1 µL genomic DNA template (about 20 ng), 2 µL forward and reverse primers (each at 5 pmol/µL), 15 µL of 2 × Taq PCR Master Mix, and 10 µL sterile ddH_2_O. The PCR cycling parameters comprised an initial denaturation at 95 °C for 5 minutes, followed by 35 amplification cycles at 95 °C (30 seconds), 50 °C (30 seconds, annealing), and 72 °C (1 minute, extension). The PCR products were verified via electrophoresis on a 1% (w/v) agarose gel.

Sequencing of PCR amplicons was carried out using an ABI 3730xL DNA Analyzer by Huayu Gene (Wuhan, China). Forward and reverse chromatograms were assembled with BioEdit v7.0.9 (Hall 1999), and the resulting sequences were subsequently aligned against the NCBI nucleotide (nt) databases using the BLAST tool to identify closely related sequences. All novel sequences generated in this study have been deposited in GenBank (Table 1).

**Table 1.** Taxa, vouchers, locations, and GenBank accession numbers of DNA sequences used in this study.

Taxon	Voucher	Locality	GenBank accession Nos.				References
			28S	ITS	*TEF*1	*RPB*2	
*Buchwaldoboletus lignicola*	–	–	–	MH234512	–	–	Unpublished
*Buchwaldoboletus lignicola*	–	Italy	—	HM003619	–	–	Unpublished
*Buchwaldoboletus lignicola*	–	Sweden	–	HM003618	–	–	Unpublished
*Buchwaldoboletus lignicola*	–	United Kingdom	–	HM003617	–	–	Unpublished
*Buchwaldoboletus lignicola*	3533	–	–	KM248950	–	–	Unpublished
*Buchwaldoboletus lignicola*	KA14-0711	South Korea	–	MH170896	–	–	Jo et al. (2019)
*Buchwaldoboletus lignicola*	KA14-0907	South Korea	–	MH170897	–	–	Jo et al. (2019)
*Buchwaldoboletus lignicola*	KM157323	–	–	GQ981493	–	–	Bidartondo et al. (2009)
*Buchwaldoboletus lignicola*	HKAS76674	Heilongjiang, NE China	KF112350	—	KF112277	KF112819	Wu et al. (2016)
*Buchwaldoboletus lignicola*	HKAS84904	Germany	KT990538	—	KT990740	KT990377	Wu et al. (2016)
***Buchwaldoboletus lignicola***	**N.K. Zeng4946 (FHMU5579)**	**Hainan, southern China**	**PV848889**	**PV848904**	**PV871779**	–	**This study**
*Buchwaldoboletus lignicola*	Pul1	Germany	JQ326997	—	JQ327040	—	Halling et al. (2012)
*Buchwaldoboletus lignicola*	VDKO1140	Belgium	—	—	MH614710	MH614756	Vadthanarat et al. (2019)
*Buchwaldoboletus* sp.	JLF_X22	USA	–	KU144820	–	–	Frank et al. (2020)
***Buchwaldoboletus xylophilus***	**M-474 (FHMU9090)**	**Yunnan, SW China**	**PV848890**	**PV848905**	**PV871778**	–	**This study**
***Buchwaldoboletus xylophilus***	**ZCX-YS-01 (FHMU11666)**	**Yunnan, SW China**	**PV848891**	**PV848906**	**PV871770**	**PV877226**	**This study**
***Buchwaldoboletus xylophilus***	**ZCX-YS-02 (FHMU11667)**	**Yunnan, SW China**	**PV848892**	**PV848907**	**PV871769**	**PV877227**	**This study**
***Buchwaldoboletus xylophilus***	**ZCX-YS-03 (FHMU11668)**	**Yunnan, SW China**	**PV848893**	**PV848908**	**PV871771**	**PV877228**	**This study**
***Buchwaldoboletus xylophilus***	**ZCX-ZP-01 (FHMU11669)**	**Yunnan, SW China**	**PV848894**	**PV848909**	**PV871772**	**PV877229**	**This study**
***Buchwaldoboletus xylophilus***	**ZCX-ZP-02 (FHMU11670)**	**Yunnan, SW China**	**PV848895**	**PV848910**	**PV871773**	**PV877230**	**This study**
***Buchwaldoboletus xylophilus***	**ZCX-ZP-03 (FHMU11671)**	**Yunnan, SW China**	**PV848896**	**PV848911**	**PV871774**	**PV877231**	**This study**
***Buchwaldoboletus xylophilus***	**ZFN-CLF (FHMU11672)**	**Yunnan, SW China**	**PV848897**	**PV848912**	**PV871775**	–	**This study**
*Buchwaldoboletus xylophilus*	FHMU5930	Yunnan, SW China	MW783417	MW783439	MW897330	MW820939	Xie et al. (2021)
*Buchwaldoboletus xylophilus*	FHMU5930-1	Yunnan, SW China	MW783418	MW783440	MW897331	MW820940	Xie et al. (2021)
*Buchwaldoboletus xylophilus*	FHMU5931	Yunnan, SW China	MW783419	MW783441	MW897332	MW820941	Xie et al. (2021)
*Buchwaldoboletus xylophilus*	FHMU5931-1	Yunnan, SW China	MW783420	MW783442	MW897333	MW820942	Xie et al. (2021)
*Buchwaldoboletus xylophilus*	FHMU5932	Yunnan, SW China	MW783421	MW783443	MW897334	MW820943	Xie et al. (2021)
*Buchwaldoboletus xylophilus*	FHMU5932-1	Yunnan, SW China	MW783422	MW783444	MW897335	MW820944	Xie et al. (2021)
*Buchwaldoboletus xylophilus*	FHMU5933	Yunnan, SW China	MW783423	MW783445	MW897336	MW820945	Xie et al. (2021)
*Buchwaldoboletus xylophilus*	FHMU5933-1	Yunnan, SW China	MW783424	MW783446	MW897337	MW820946	Xie et al. (2021)
*Buchwaldoboletus xylophilus*	X.H. Deng1 (FHMU5848)	Hainan, southern China	MW783425	—	MW897338	MW820947	Xie et al. (2021)
*Buchwaldoboletus xylophilus*	X.H. Deng2 (FHMU5849)	Hainan, southern China	MW783426	—	MW897339	MW820948	Xie et al. (2021)
Chalciporus aff. piperatus	HKAS50214	Yunnan, SW China	JQ928621	–	JQ928610	—	Hosen et al. (2012)
*Chalciporus africanus*	JD0517	Cameroon	—	—	KT824029	KT823996	Raspé et al. (2016)
*Chalciporus amarellus*	DS4640-3	Germany	KF030283	—	KF030440	—	Nuhn et al. (2013)
***Chalciporus aurantiolepidotus***	**N.K. Zeng7075 (FHMU7049), holotype**	**Hainan, southern China**	**PV910623**	**PV910636**	**PV891372**	**PV915955**	**This study**
***Chalciporus aurantiolepidotus***	**N.K. Zeng7076 (FHMU7056)**	**Hainan, southern China**	**PV910624**	**PV910637**	**PV891371**	**PV915956**	**This study**
***Chalciporus aurantiolepidotus***	**N.K. Zeng7352 (FHMU7472)**	**Hainan, southern China**	**PV910625**	**PV910638**	**PV891367**	–	**This study**
***Chalciporus brunneus***	**N.K. Zeng8522 (FHMU8246), holotype**	**Hainan, southern China**	**PV910626**	**PV910639**	**PV891368**	**PV927291**	**This study**
***Chalciporus brunneus***	**N.K. Zeng8522-1 (FHMU11617)**	**Hainan, southern China**	**PV910627**	**PV910640**	**PV891369**	**PV927292**	**This study**
***Chalciporus brunneus***	**N.K. Zeng8522-2 (FHMU11547)**	**Hainan, southern China**	**PV910628**	**PV910641**	**PV891370**	**PV927293**	**This study**
***Chalciporus brunneus***	**N.K. Zeng8522-3 (FHMU11548)**	**Hainan, southern China**	**PV910629**	**PV910642**	**PV891373**	**PV927294**	**This study**
*Chalciporus citrinoaurantius*	GDGM44776	Hunan, central China	MZ157131	OM877502	MZ165617	MZ165608	Zhang et al. (2017)
*Chalciporus citrinoaurantius*	GDGM44480	Hunan, central China	MZ157128	OM877499	MZ165614	MZ165605	Zhang et al. (2017)
*Chalciporus citrinoaurantius*	GDGM44481	Hunan, central China	MZ157129	OM877500	MZ165615	MZ165606	Zhang et al. (2017)
*Chalciporus citrinoaurantius*	GDGM44717	Hunan, central China	MZ157130	OM877501	MZ165616	MZ165607	Zhang et al. (2017)
*Chalciporus hainanensis*	GDGM44464	Hainan, southern China	MZ157127	OM877505	MZ165612	MZ165604	Zhang et al. (2017)
*Chalciporus hainanensis*	GDGM46161	Hainan, southern China	MZ157126	—	MZ165613	MZ165609	Zhang et al. (2017)
*Chalciporus hainanensis*	S. Jiang81 (FHMU4573)	Hainan, southern China	MW917176	—	MW925933	—	Xu et al. (2021)
***Chalciporus hainanensis***	**N.K. Zeng8211 (FHMU9951)**	**Fujian, SE China**	**PV848898**	–	–	–	**This study**
*Chalciporus perezsilvae*	MEXU-HO 30438	Mexico	OR421572	OR421044	—	—	Ayala-Vásquez et al. (2023)
*Chalciporus piedracanteadensis*	MEXU-HO 30436	Mexico	OR421570	OR421042	—	—	Ayala-Vásquez et al. (2023)
*Chalciporus piedracanteadensis*	MEXU-HO 30437	Mexico	OR421571	OR421043	—	—	Ayala-Vásquez et al. (2023)
*Chalciporus piperatus*	HKAS84882	Germany	KT990562	—	KT990758	KT990397	Wu et al. (2016)
*Chalciporus piperatus*	BJTC FM2220	Shanxi, northern China	OR655148	OR655148	OR659985	OR659936	Mao et al. (2023)
*Chalciporus pseudorubinellus*	4302	USA	KF030284	—	KF030441	—	Nuhn et al. (2013)
*Chalciporus pseudorubinellus*	BN07	NH, USA	KF030286	—	—	—	Nuhn et al. (2013)
*Chalciporus pseudorubinellus*	DS61207	NY, USA	KF030287	–	KF030441	—	Nuhn et al. (2013)
*Chalciporus radiatus*	GDGM43285	Hunan, central China	KP871800	KP871804	MZ165610	—	Zhang et al. (2016); Zhang et al. (2017)
*Chalciporus radiatus*	GDGM43305	Guangdong, southern China	KP871802	—	—	—	Zhang et al. (2016)
*Chalciporus radiatus*	GDGM50080	Hunan, central China	KP871801	KP871806	MZ165611	—	Zhang et al. (2016); Zhang et al. (2017)
***Chalciporus radiatus***	**N.K. Zeng10292 (FHMU8509)**	**Zhejiang, eastern China**	**PV848899**	**PV848913**	**PV871776**	**PV877232**	**This study**
***Chalciporus radiatus***	**N.K. Zeng10296 (FHMU8989)**	**Zhejiang, eastern China**	**PV848900**	–	**PV871777**	**PV877233**	**This study**
*Chalciporus radiatus*	N.K. Zeng1379 (FHMU930)	Fujian, SE China	MH879710	—	MH879738	—	Chai et al. (2019)
*Chalciporus radiatus*	N.K. Zeng1414 (FHMU959)	Fujian, SE China	MH879711	—	MH879739	—	Chai et al. (2019)
*Chalciporus radiatus*	N.K. Zeng1808 (FHMU 2494)	Hainan, southern China	—	—	MH879737	—	Chai et al. (2019)
***Chalciporus roseus***	**N.K. Zeng8516 (FHMU7888), holotype**	**Hainan, southern China**	**PV910630**	**PV910643**	**PV891611**	–	**This study**
***Chalciporus roseus***	**N.K. Zeng8516-1 (FHMU11614)**	**Hainan, southern China**	**PV910631**	**PV910644**	**PV891612**	–	**This study**
***Chalciporus roseus***	**N.K. Zeng8516-2 (FHMU11615)**	**Hainan, southern China**	**PV910632**	**PV910645**	**PV915952**	–	**This study**
*Chalciporus rubinelloides*	HKAS57362	Yunnan, SW China	KT990563	—	KT990759	KT990398	Wu et al. (2016)
*Chalciporus rubinelloides*	HKAS58728	Yunnan, SW China	KT990564	—	KT990760	KT990399	Wu et al. (2016)
*Chalciporus rubinelloides*	HKAS74952	Yunnan, SW China	KT990565	—	KT990761	KT990400	Wu et al. (2016)
*Chalciporus rubinelloides*	HKAS75034	Yunnan, SW China	KT990566	—	—	—	Wu et al. (2016)
*Chalciporus rubinellus*	191/81	USA	EU685106	—	—	—	Desjardin et al. (2009)
*Chalciporus rubrostipitatus*	ZGCSN153	India	OQ193026	OQ225690	—	OQ993343	Nanu and Kumar (2024)
*Chalciporus rubrostipitatus*	ZGCSN160	India	—	OQ231504	—	—	Nanu and Kumar (2024)
*Chalciporus sinensis*	N.K. Zeng4478 (FHMU4701)	Hainan, southern China	MW917171	—	MW925928	MW925934	Xu et al. (2021)
*Chalciporus sinensis*	N.K. Zeng4479 (FHMU4691)	Hainan, southern China	MW917172	—	MW925929	MW925935	Xu et al. (2021)
*Chalciporus* sp.	GDGM43250	Guangdong, southern China	MZ157125	—	—	—	Zhang et al. (2017)
*Chalciporus* sp.	HKAS53400	Hunan, central China	KF112352	—	KF112279	KF112821	Wu et al. (2023)
*Chalciporus* sp.	OR0363	Thailand	—	—	MH645594	MH645602	Vadthanarat et al. (2019)
*Chalciporus* sp.	X.T. Zhu134 (FHMU2721)	Yunnan, SW China	MW917175	—	MW925932	—	Xu et al. (2021)
*Chalciporus vulparius*	N.K. Zeng4978 (FHMU5554)	Hainan, southern China	MW917173	—	MW925930	MW925936	Xu et al. (2021)
*Chalciporus vulparius*	N.K. Zeng4979 (FHMU5560)	Hainan, southern China	MW917174	—	MW925931	MW925937	Xu et al. (2021)
*Paxillus obscurosporus*	Po1	Germany	AY177256	–	KF030442	–	Nuhn et al. (2013); Wu et al. (2016)
*Paxillus vernalis*	AFTOL-ID 715	–	AY645059	DQ647827	DQ457629	–	Binder and Hibbett (2006); Matheny et al. (2006)
*Pseudophylloporus baishanzuensis*	N.K. Zeng7702 (FHMU7694)	Zhejiang, eastern China	PQ330210	–	PQ330110	PQ330114	Qin et al. (2024)
*Pseudophylloporus baishanzuensis*	N.K. Zeng7703 (FHMU7695)	Zhejiang, eastern China	PQ330211	–	PQ330111	PQ330115	Qin et al. (2024)
*Pseudophylloporus baishanzuensis*	N.K. Zeng7705 (FHMU7696)	Zhejiang, eastern China	PQ330212	–	PQ330112	PQ330116	Qin et al. (2024)
*Pseudophylloporus baishanzuensis*	N.K. Zeng7746 (FHMU7697)	Zhejiang, eastern China	PQ330213	–	PQ330113	PQ330117	Qin et al. (2024)
***Pseudophylloporus castaneus***	**FC330727230331 (ZJMR330727230331), holotype**	**Zhejiang, eastern China**	**PV910633**	**PV910646**	–	–	**This study**
***Pseudophylloporus castaneus***	**FC330727230331-1 (FHMU11549)**	**Zhejiang, eastern China**	**PV910634**	**PV910647**	**PV915953**	–	**This study**
***Pseudophylloporus castaneus***	**FC330727230331-2 (FHMU11550)**	**Zhejiang, eastern China**	**PV910635**	**PV910648**	**PV915954**	–	**This study**

GenBank numbers in bold indicate the newly generated sequences; SE, Southeastern; SW, Southwest; NE, Northeast.

### ﻿Dataset assembly

A total of 85 novel DNA sequences (including 25 for 28S, 23 for ITS, 23 for *TEF*1, and 14 for *RPB*2) were obtained from 25 collections. These sequences, along with reference sequences retrieved from GenBank and previously published literature (Table 1), were combined to construct a concatenated dataset comprising the 28S, ITS, *TEF*1, and *RPB*2 gene regions. Reference sequences were selected based on the following criteria: (i) preference for sequences derived from type material when available; (ii) inclusion of high-quality sequences with multiple gene fragments from published studies; and (iii) ensuring broad taxonomic and geographic representation of Chalciporoideae. Following Wu et al. (2016), *Paxillus vernalis* Watling and *P. obscurosporus* C. Hahn were selected as outgroup taxa. To detect potential phylogenetic conflicts among the four gene regions within the combined dataset, single-gene analyses were performed separately for the 28S, ITS, *TEF*1, and *RPB*2 sequences. Results indicated no significant incongruence among these gene trees. Subsequently, individual gene sequences were aligned using MUSCLE v3.6 (Edgar 2004) and manually refined using BioEdit software (Hall 1999). Finally, aligned sequences were concatenated utilizing Phyutility version 2.2 (Smith and Dunn 2008) for downstream phylogenetic analysis.

### ﻿Phylogenetic analyses

For the phylogenetic reconstruction based on the concatenated dataset (28S + ITS + *TEF*1 + *RPB*2), both Maximum Likelihood (ML) and Bayesian Inference (BI) methodologies were utilized. ML analyses and associated bootstrap tests were executed using RAxML v7.2.6 (Stamatakis 2006). The GTRGAMMA substitution model was selected for ML analyses, while all other settings remained at default values. Support values were estimated via nonparametric bootstrap analysis with 1000 replicates. Bayesian analyses employed Markov Chain Monte Carlo (MCMC) sampling using MrBayes v3.1 (Huelsenbeck and Ronquist 2005; Miller et al. 2011). Two parallel MCMC runs, each comprising four simultaneous chains, were carried out. Trees were sampled every 100 generations. Parameter settings followed default options, guided by MrModeltest v2.3 recommendations (Nylander 2004). The initial 25% of sampled trees were excluded as burn-in, and the remaining trees were used to generate a majority-rule consensus tree, with posterior probability (PP) values calculated accordingly.

## ﻿Results

### ﻿Molecular data

**Figure 1.**
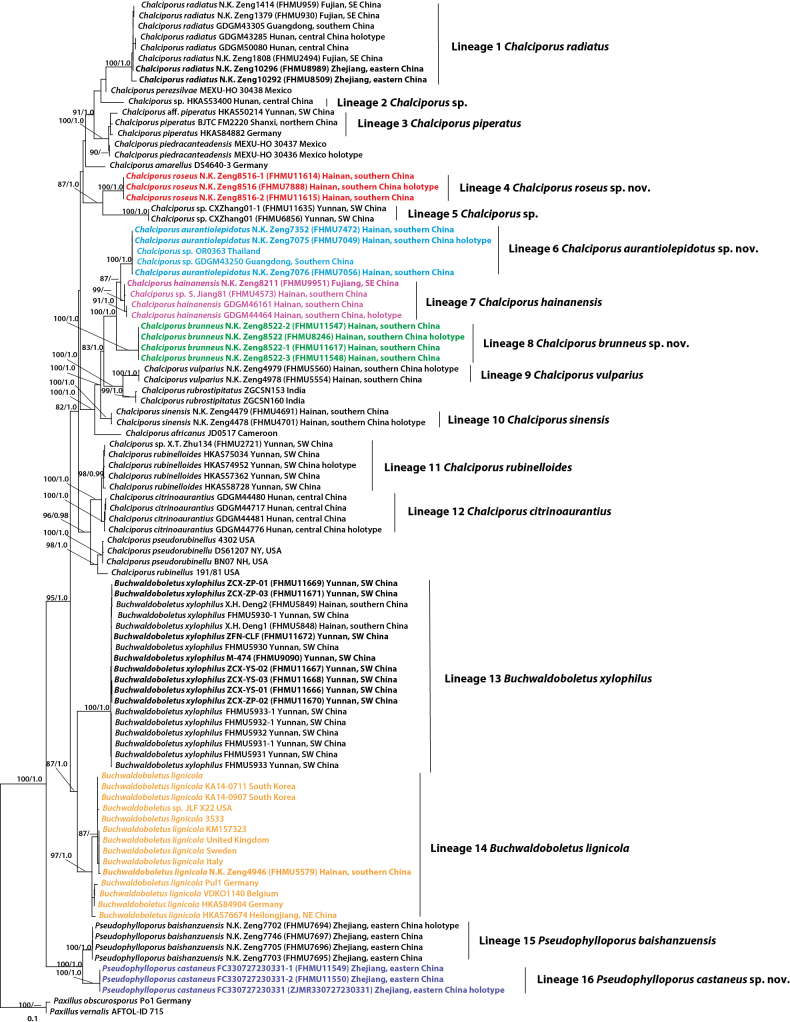
Phylogram of Chalciporoideae inferred from multilocus (rDNA 28S, ITS, *TEF*1, and *RPB*2) dataset using RAxML. RAxML bootstrap percentages (BS ≥ 70%) and Bayesian posterior probabilities (PP ≥ 0.95) are indicated above or below the branches as BS/PP. Note. SE = southeastern China; SW = southwestern China; NE = northeastern China.

The dataset, combining 28S, ITS, *TEF*1, and *RPB*2, included 95 sequences with 2141 nucleotide positions (28S: 652 bp; ITS: 500 bp; *TEF*1: 373 bp; *RPB*2: 616 bp). Of these, 588 sites were variable and 533 were parsimony-informative. Bayesian analyses for the multi-gene dataset were run for 30 million generations, reaching convergence with an average standard deviation of split frequencies at 0.000499. The optimal nucleotide substitution models for each gene partition were identified as SYM + G for 28S, ITS, *TEF*1, and *RPB*2. The Bayesian analyses produced topologies identical to those of the ML analysis, with slight differences in statistical support (Fig. 1). The collections of Chalciporoideae from China were grouped into 16 independent species-level lineages (Fig. 1).

## ﻿Taxonomy

### ﻿Key to genera of Chalciporoideae

**Table:** 

1	Hymenophore lamellate, clamp connections present	*** Pseudophylloporus ***
–	Hymenophore poroid, clamp connections absent	**2**
2	Pileus surface tomentose or pulverulent, yellow to brownish; pore surface initially light yellow to ochraceous yelow	*** Buchwaldoboletus ***
–	Pileus surface glabrous to obscurely subtomentose, pinkish-red to reddish-brown; pore surface pinkish red to reddish brown, yellow	*** Chalciporus ***

#### ﻿ Buchwaldoboletus

Taxon classificationFungiBoletalesBoletaceae

Pilát, Friesia 9 (1–2): 217, 1969

745E82B0-DDC4-5A12-BD2B-C7FF00AB7F61

Buchwaldoboletus , typified by B. lignicola, is mainly characterized by its saprotrophic and lignicolous lifestyle, a dry, pulverulent to tomentose pileus, yellow to golden hymenophore changing blue when injured, and an interwoven pileipellis (Pilát 1969; Ortiz-Santana and Both 2011; Wu et al. 2016). Until now, two species, viz. B. lignicola and B. xylophilus have been confirmed to occur in China (Wu et al. 2016; Xie et al. 2021).

#### ﻿ Buchwaldoboletus lignicola

Taxon classificationFungiBoletalesBoletaceae

(Kallenb.) Pilát, Friesia. 9 (1–2): 217, 1969

38BF004A-9FF6-5EF8-890F-1AE7494656C2

327206

Figs 2a, b, 3

Boletus lignicola Kallenb., Die Pilze Mitteleuropas, Band 1, Die Röhrlinge (Boletaceae): 57, 1929.
Xerocomus lignicola (Kallenb.) Singer, Annales Mycologici 40: 43, 1942.
Phlebopus lignicola (Kallenb.) M.M. Moser ex Groves, Mycologia 54: 320, 1962.
Pulveroboletus lignicola (Kallenb.) E.A. Dick & Snell, Mycologia 57: 451, 1965.
Gyrodon lignicola (Kallenb.) Heinem., Bulletin du Jardin Botanique de l’État à Bruxelles 21: 238, 1951.

##### Description.

***Basidiomata*** small-sized. Pileus 3–4 cm in diameter, subhemispherical to convex; surface dry, ﬁnely tomentose, orangish-yellow to golden-yellow (4A6–5A7); context 0.4–0.6 cm in thickness in the center of the pileus, white (1A1) to yellowish (1A2), unchanging in color when bruised. ***Hymenophore*** poroid, slightly decurrent; pores angular to roundish, yellow, changing greenish-blue when bruised; tubes 0.1–0.3 cm long, yellow, changing greenish-blue when bruised. ***Stipe*** 3–5 × 1–2 cm, central, solid, subcylindrical, surface tomentose, upper part pale yellow to yellow (3A4–3A5), lower part orangish-brown to ochre (5C5–6C6); context white (1A1) to yellowish (1A2), unchanging in color when bruised; basal mycelium white. ***Odor*** indistinct. ***Taste*** mild.

**Figure 2.**
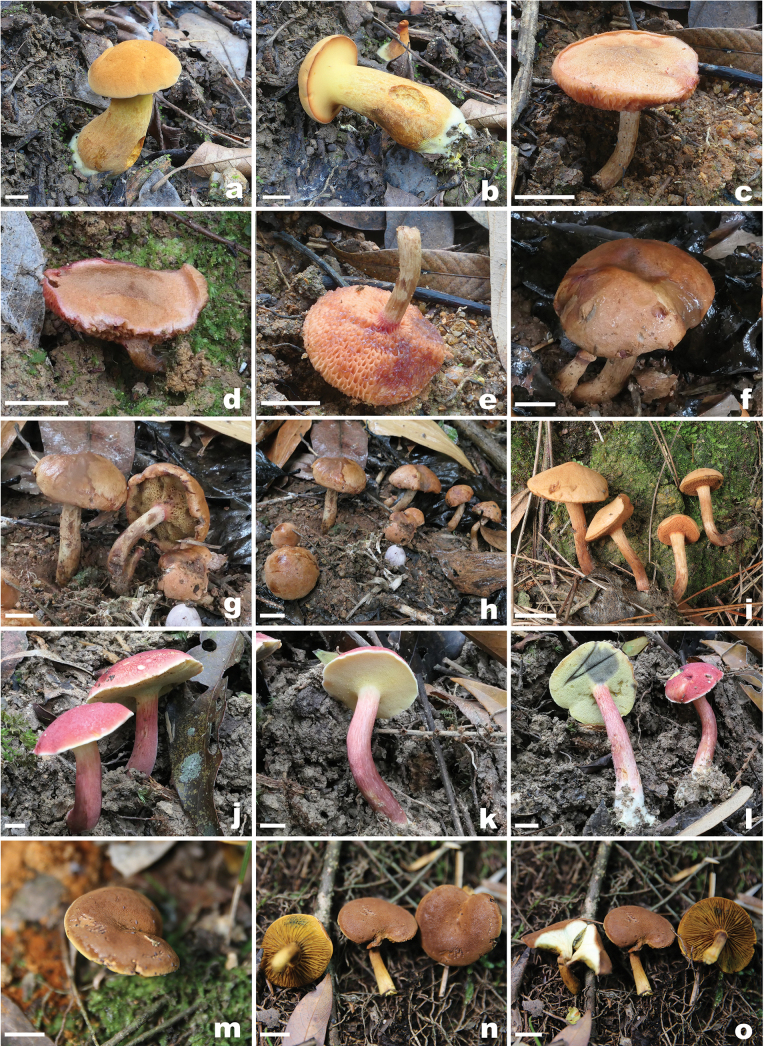
Basidiomata of fungi in the subfamily Chalciporoideae a, b. *Buchwaldoboletus lignicola* (FHMU5579); c–e. *Chalciporus aurantiolepidotus* (c, e FHMU7049, holotype; d FHMU7472); f–h. *C. brunneus* (FHMU8246, holotype); i. *C. hainanensis* (FHMU9951); j–l. *C. roseus* (FHMU7888, holotype); m–o. *Pseudophylloporus castaneus* (ZJMR330727230331, holotype). Scale bars: 1 cm. a–l. photos by N.K. Zeng; m–o. photos by J.B. Pu.

***Basidiospores*** [40/2/1] (6)6.5–9 × (3)3.5–4 μm, Q= (1.5)1.75–3.0, Qm = 2.30 ± 0.32, yellowish brown in KOH, elongated to cylindrical, slightly thick-walled (0.8–1 µm), smooth under the light microscope. ***Basidia*** 14–29 × 5–8 μm, clavate, slightly thick-walled (up to 1 µm), 4-spored, hyaline to pale yellow in KOH; sterigmata 2–5 μm in length. ***Cystidia*** 20–35 × 5–7 μm, fusiform or subfusiform, slightly thick-walled (up to 1 μm), pale yellow in KOH. ***Hymenophoral trama*** boletoid, colorless to yellowish in KOH, thin- to slightly thick-walled (up to 0.5 μm), 6–12 μm wide. ***Pileipellis*** an intricate trichoderm 400–550 μm in thickness, composed of hyaline to pale yellow in KOH, slightly thick-walled (up to 1 μm) hyphae; terminal cells 9–37 × 3–7 μm, clavate to subcylindrical or cystidioid. ***Pileus trama*** made up of hyphae 2–9 μm in diameter, thick-walled (up to 1.5 μm), yellow in KOH. ***Stipitipellis*** a trichoderm-like structure 160–200 μm thick, composed of pale yellow in KOH, thin-walled hyphae; terminal cells 18–27× 5–8 μm, clavate or subcylindrical. ***Stipe trama*** composed of parallel hyphae 2–16 μm in diameter, cylindrical, thin-walled, yellow in KOH. ***Clamp connections*** absent in all tissues.

**Figure 3.**
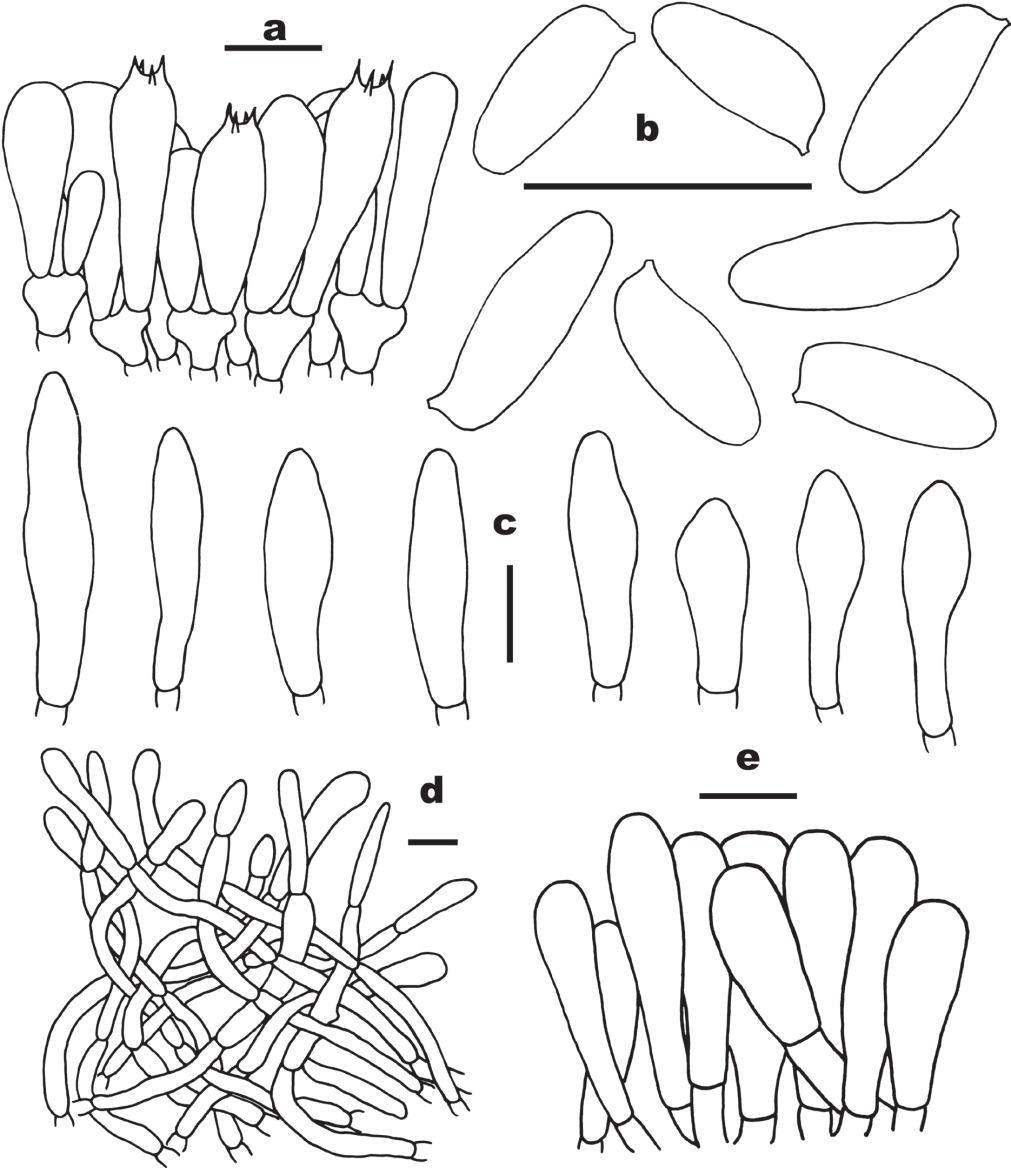
Microscopic features of *Buchwaldoboletus lignicola* (FHMU5579). a. Basidia; b. Basidiospores; c. Cystidia; d. Pileipellis; e. Stipitipellis. Scale bars: 10 µm. Drawings by X.N. Li.

##### Habitat.

Solitary on the ground in broadleaf forest.

##### Known distribution.

Southern (Hainan Province) and northeastern China (Heilongjiang Province); Europe, North America, Nepal, Korea, and Indonesia (Kallenbach 1929; Aryal and Budathoki 2013; Wu et al. 2016; Venturella 2017; Jo et al. 2019; Putra et al. 2025).

##### Material examined.

China • Hainan Province, Changjiang County, Bawangling of Hainan Tropical Rainforest National Park, elev. 650 m, 2 September 2020, N.K. Zeng4946 (FHMU5579).

##### Notes.

*Buchwaldoboletus lignicola* was originally described from Germany by Kallenbach (1929). In China, it was reported from the northeastern region of the country (Wu et al. 2016). In the present study, it was also found to be distributed in Hainan Province, southern China. The species is characterized by a dry, tomentose, orangish-yellow to golden-yellow pileus, a yellow hymenophore bruising greenish-blue, and an intricate trichodermal pileipellis. Based on our specimen, a diagnostic feature described by Wu et al. (2016), viz., doliform to subglobose cells often forming chains in the pileipellis, was not observed. Such cellular morphology has not been reported in other recent descriptions of *B. lignicola*, including specimens from Korea (Jo et al. 2019).

#### ﻿ Buchwaldoboletus xylophilus

Taxon classificationFungiBoletalesBoletaceae

(Petch) Both & B. Ortiz, Bull. Buffalo Soc. Nat. Sci. 40: 3, 2011

E06F4846-3D02-5412-B667-C070E6F2B61A

545884

Boletus xylophilus Petch, Ann. Roy. Bot. Gard. Peradeniya 7 (4): 283, 1922.
Gyrodon xylophilus (Petch) Heinem. & Rammeloo, Bull. Jard. Bot. Natl. Belg. 53 (1–2): 295, 1983.
Pulveroboletus xylophilus (Petch) Singer, in Singer, Araujo & Ivory, Beih. Nova Hedwigia 77: 98, 1983.

##### Known distribution.

Southwestern (Yunnan Province) and southern China (Hainan Province and Hong Kong); Sri Lanka, Malaysia, India, and the Philippines (Pegler 1986; Corner 1972; Ortiz-Santana and Both 2011; Xie et al. 2021; Nanu and Kumar 2022).

##### Holotype.

Petch 5812 (K) (Sri Lanka) (non vidi).

##### Materials examined.

China • Yunnan Province, Xishuangbanna, cultivated at the Yunnan Institute of Tropical Crops, December 2023, M-474 (FHMU9090); • same location and date, ZCX-YS-01 (FHMU11666); • same location and date, ZCX-YS-02 (FHMU11667); • same location and date, ZCX-YS-03 (FHMU11668); • same location and date, ZCX-ZP-01 (FHMU11669); • same location and date, ZCX-ZP-02 (FHMU11670); • same location and date, ZCX-ZP-03 (FHMU11671); • same location and date, ZFN-CLF (FHMU11672).

##### Notes.

*Buchwaldoboletus xylophilus* was originally described from Sri Lanka by Petch (1922). It was also reported from southwestern and southern China (Xie et al. 2021; Mao et al. 2023). Illustrations and a full description of the species have been provided by Ortiz-Santana and Both 2011 and Xie et al. (2021).

### ﻿Key to accepted *Buchwaldoboletus* species in China

**Table:** 

1	Pileus orangish-yellow; context unchanging in color when bruised; basidiospores elongated, measuring 6.5–9 × 3.5–4 μm, Qm = 2.30 ± 0.32	***B. lignicola***
–	Pileus yellowish-brown; context turning blue in color when bruised; basidiospores subglobose to short-ellipsoid, measuring 4.5–6 × 3–4.5 µm, Qm = 1.38 ± 0.23	***B. xylophilus***

#### ﻿ Chalciporus

Taxon classificationFungiBoletalesBoletaceae

Bataille, Bull. Soc. Hist. Nat. Doubs 15: 39, 1908

8499E262-86A0-5B88-9D7E-F84ACB9BAB8D

Chalciporus , typified by C. piperatus, was originally established to accommodate species with a small basidioma, a reddish hymenophore, and smooth basidiospores (Moreno and García-Bona 1976; Pegler and Young 1981; Pegler 1983; Baroni and Both 1991; Gómez 1996; Halling et al. 2004; Klofac and Krisai-Greilhuber 2006). Besides the three new Chalciporus species revealed in the present study, seven species of the genus have been confirmed to occur in China (Wu et al. 2016; Zhang et al. 2016, 2017; Deng et al. 2018; Chai et al. 2019; Xu et al. 2021; Mao et al. 2023).

#### ﻿ Chalciporus aurantiolepidotus

Taxon classificationFungiBoletalesBoletaceae

N.K. Zeng & X. Zhang sp. nov.

24AF8F34-9D19-5219-9B6B-B33BA5FC2E3D

860039

Figs 2c–e, 4

##### Etymology.

Latin “*aurantiolepidotus*”, referring to the orange squamules on the pileal surface.

##### Holotype.

China • Hainan Province, Changjiang County, Bawangling of Hainan Tropical Rainforest National Park, alt. 650 m, 19.1°N, 109.2°E, 8 May 2022, N.K. Zeng7075 (FHMU7049).

##### Diagnosis.

Differs from closest species of *Chalciporus* by a very small basidioma, a dry pileus covered with orange to reddish squamules, a reddish-orange to reddish hymenophore, and a trichodermal pileipellis.

##### Description.

***Basidiomata*** very small-sized. ***Pileus*** 2–2.5 cm in diameter, subhemispherical to convex when young, plano-convex to applanate when mature, margin occasionally upturned; surface dry, covered with orange (6B4) to reddish (6B3) squamules; context 0.2–0.3 cm in thickness in the center of the pileus, white (1A1), unchanging in color when bruised. ***Hymenophore*** poroid, slightly decurrent; pores angular, 0.1–0.25 cm wide, reddish-orange (7A4) to reddish (8A5), unchanging in color when bruised; tubes 0.2–0.3 cm long, reddish (8A5), unchanging in color when bruised. ***Stipe*** 1.6–1.7 × 0.3 cm, central, solid, subcylindrical; surface pale brown, with distinctly longitudinal striations; context white (1A1), unchanging in color when bruised; basal mycelium white. ***Odor*** indistinct. ***Taste*** mild.

**Figure 4.**
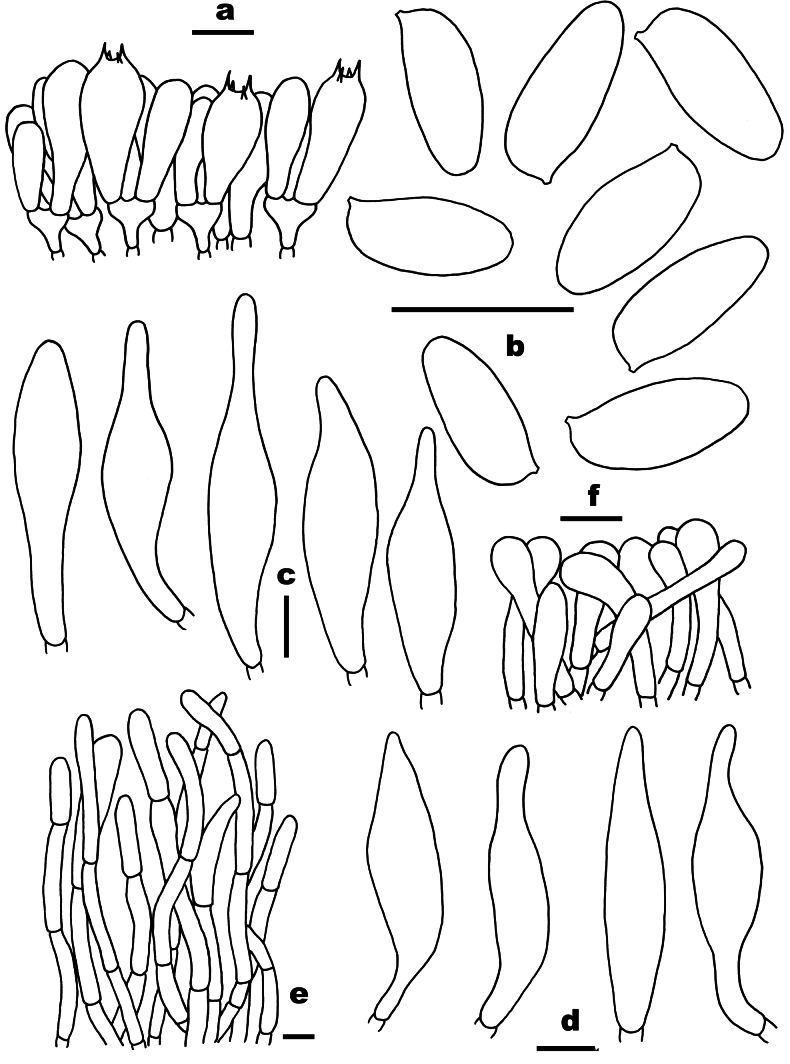
Microscopic features of *Chalciporus aurantiolepidotus* (FHMU7049, holotype). a. Basidia; b. Basidiospores; c. Cheilocystidia; d. Pleurocystidia; e. Pileipellis; f. Stipitipellis. Scale bars: 10 µm. Drawings by X.N. Li.

***Basidiospores*** [60/8/3] (8)8.5–11(12) × 4–5 μm, Q= (1.6)1.7–2.5(3.0), Q_m_ = 2.22 ± 0.28, yellowish-brown in KOH, elongated to cylindrical, slightly thick-walled (0.8–1 µm), smooth under the light microscope. ***Basidia*** 18–30 × 8–12 μm, clavate, slightly thick-walled (up to 1 µm), 4-spored, colorless to pale yellow in KOH; sterigmata 2–5.5 μm in length. ***Cheilocystidia*** 38–63 × 8–12 μm, fusiform or subfusiform, slightly thick-walled (up to 1 μm), colorless to pale yellow in KOH. ***Pleurocystidia*** 41–61 × 9–13 μm, fusiform or subfusiform, slightly thick-walled (up to 1 μm), pale yellow in KOH. ***Hymenophoral trama*** boletoid, colorless to yellowish in KOH, thin- to slightly thick-walled (up to 0.5 μm), 5–10 μm wide. ***Pileipellis*** a trichoderm 250–300 μm in thickness, composed of pale yellow in KOH, slightly thick-walled (up to 1 μm) hyphae; terminal cells 20–61 × 5–9 μm, clavate to subcylindrical or cystidioid. ***Pileus trama*** made up of hyphae 5–19 μm in diameter, thick-walled (up to 1.5 μm), yellow in KOH. ***Stipitipellis*** a trichoderm-like structure 150–200 μm thick, composed of hyaline to pale yellow in KOH, thin-walled hyphae; terminal cells 15–30× 4–7 μm, clavate or subcylindrical, occasionally subfusiform. ***Stipe trama*** composed of parallel hyphae 3–7 μm in diameter, cylindrical, thin-walled, yellow in KOH. ***Clamp connections*** absent in all tissues.

##### Habitat.

Solitary or scattered on the ground in forests dominated by fagaceous trees (*Lithocarpus* spp.).

##### Known distribution.

Southern China (Hainan Province), probably Guangdong Province, and Thailand (Fig. 1).

##### Additional materials examined.

China • Hainan Province: Changjiang County, Bawangling of Hainan Tropical Rainforest National Park, alt. 650 m, 19.1°N, 109.2°E, 18 May 2022, N.K. Zeng7076 (FHMU7056; ZJMR330727230331); • Baisha County, Yinggeling of Hainan Tropical Rainforest National Park, alt. 650 m, 19.2°N, 109.5°E, 20 July 2022, N.K. Zeng7352 (FHMU7472).

##### Notes.

*Chalciporus aurantiolepidotus* is phylogenetically related and morphologically similar to *C. brunneus*, *C. hainanensis* Ming Zhang & T.H. Li, and *C. vulparius*. However, *C. brunneus* has a pale brown to brown pileus, a yellow hymenophore, a stipe usually reddish at the apex, and an intricate trichodermal pileipellis (see below); *C. hainanensis* has a light orange-brown pileus, a white context changing red when bruised, and a stipe usually reddish at the apex (see below); *C. vulparius* has a tomentose, reddish-brown to pale reddish-brown pileus, a reddish-pink hymenophore, a stipe covered with white to brown scales, and shorter basidiospores measuring 5.5–9 × 3–5 μm (Xu et al. 2021).

#### ﻿ Chalciporus brunneus

Taxon classificationFungiBoletalesBoletaceae

N.K. Zeng & X. Zhang sp. nov.

E3821C42-DAC1-563B-BE33-A4D943EDB56F

860040

Figs 2f–h, 5

##### Etymology.

Latin “*brunneus*”, referring to the pale brown to brown pileus.

**Figure 5.**
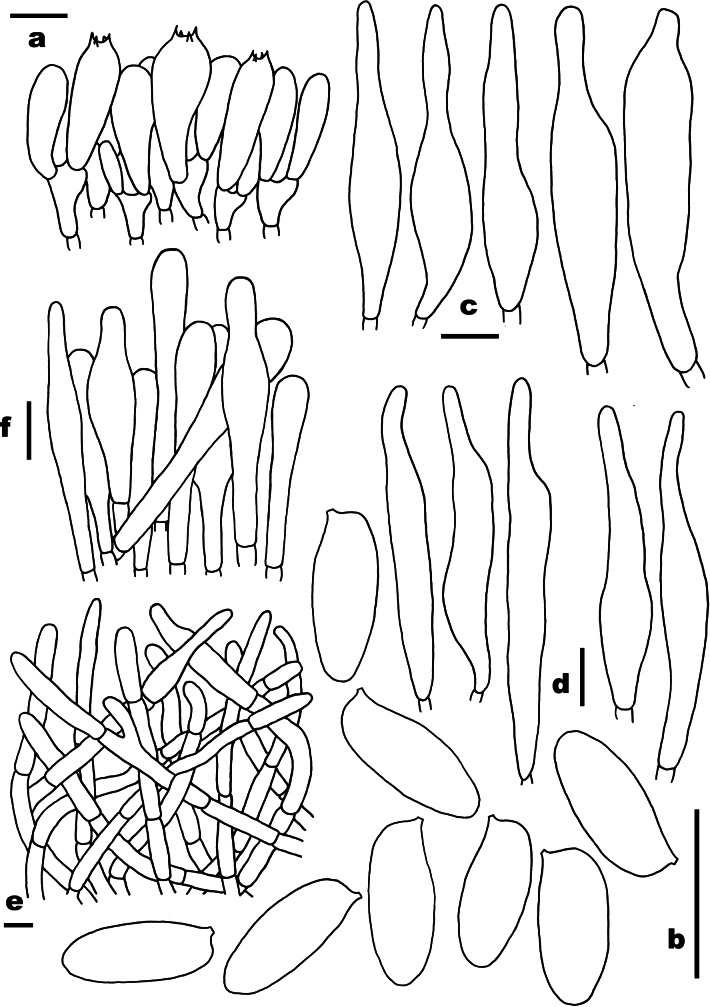
Microscopic features of *Chalciporus brunneus* (FHMU8246, holotype). a. Basidia; b. Basidiospores; c. Cheilocystidia; d. Pleurocystidia; e. Pileipellis; f. Stipitipellis. Scale bars: 10 µm. Drawings by X.N. Li.

##### Holotype.

China • Hainan Province, Changjiang County, Bawangling of Hainan Tropical Rainforest National Park, alt. 650 m, 19.2°N, 109.1°E, 13 May 2025, N.K. Zeng8522 (FHMU8246).

##### Diagnosis.

Differs from closest species of *Chalciporus* by a pale brown to brown pileus, a yellowish-white context unchanging when bruised, a yellow hymenophore, a stipe usually reddish at the apex, and an intricate trichodermal pileipellis.

##### Description.

***Basidiomata*** very small to small-sized. ***Pileus*** 1.2–3.6 cm in diameter, subhemispherical to convex or plano-convex, margin incurved; surface slightly viscid when wet, pale brown to brown (6C2–6C3); context 0.2–0.7 cm in thickness in the center of the pileus, yellowish white (1A2), unchanging in color when bruised. ***Hymenophore*** poroid, slightly decurrent; pores roundish or angular, 0.15–0.25 cm wide, yellow (5A4), unchanging in color when bruised; tubes approximately 0.3 cm long, yellowish orange (5A5), unchanging in color when bruised. ***Stipe*** 1.8–3.8 × 0.3–0.65 cm, central, solid, subcylindrical; surface smooth, usually reddish at the apex, yellowish white (1A2) in the upper part, gradually becoming brown (6B2) toward the base, with distinct reddish longitudinal striations; context yellowish white (1A2), unchanging in color when bruised; basal mycelium white. ***Odor*** indistinct. ***Taste*** mild.

***Basidiospores*** [160/8/4] (7)8–10.5(11) × 3–5 μm, Q= (1.70)1.90–3.00(3.33), Q_m_ = 2.34 ± 0.31, yellowish brown in KOH, elongated to cylindrical, slightly thick-walled (0.8–1 µm), smooth under the light microscope. ***Basidia*** 19–30 × 6–9 μm, clavate, slightly thick-walled (up to 1 µm), 4-spored, colorless to pale yellow in KOH; sterigmata 2–4 μm in length. ***Cheilocystidia*** 33–65 × 5–11 μm, fusiform or subfusiform, slightly thick-walled (up to 1 µm), colorless in KOH. ***Pleurocystidia*** 33–71 × 6–9 μm, fusiform or subfusiform, slightly thick-walled (up to 1 µm), colorless in KOH. ***Hymenophoral trama*** boletoid, colorless to yellowish in KOH, thin- to slightly thick-walled (up to 0.5 μm), 4–10 μm wide. ***Pileipellis*** an intricate trichoderm 200–360 μm in thickness, composed of hyaline in KOH, slightly thick-walled (up to 1 µm) hyphae; terminal cells 8–69 × 4–8 μm, clavate to subcylindrical or cystidioid. ***Pileus trama*** made up of hyphae 4–20 μm in diameter, slightly thick-walled (up to 1 µm), colorless in KOH. ***Stipitipellis*** a trichoderm-like structure 150–300 μm thick, composed of hyaline to pale yellow in KOH, slightly thick-walled (up to 1 µm) hyphae; terminal cells 30–60× 5–6 μm, clavate or subcylindrical, occasionally subfusiform. ***Stipe trama*** composed of parallel hyphae 3–13 μm in diameter, cylindrical, thin- to slightly thick-walled (up to 1 μm), hyaline to pale yellow in KOH. ***Clamp connections*** absent in all tissues.

##### Habitat.

Gregarious on the ground in forests dominated by fagaceous trees (*Lithocarpus* spp.).

##### Known distribution.

Southern China (Hainan Province).

##### Additional materials examined.

China • Hainan Province, Changjiang County, Bawangling of Hainan Tropical Rainforest National Park, alt. 650 m, 19.2°N, 109.1°E, 12 May 2025, N.K. Zeng8522-1 (FHMU11617); • same location and date, N.K. Zeng8522-2 (FHMU11547); • same location and date, N.K. Zeng8522-3 (FHMU11548).

##### Notes.

*Chalciporus brunneus* is phylogenetically related to *C. aurantiolepidotus* and *C. hainanensis*. However, *C. hainanensis* has a light orange-brown pileus, a white context turning red when bruised, and a reddish-orange hymenophore (see below). The morphological differences between *C. brunneus* and *C. aurantiolepidotus* have been discussed above. Morphologically, *C. brunneus* is similar to *C. sinensis*. However, *C. sinensis* has a pileus covered with brown to grey-brown scales, shorter, subglobose to ellipsoid basidiospores measuring 4–7 × 3.5–5 μm, and a trichodermial pileipellis (Xu et al. 2021).

#### ﻿ Chalciporus citrinoaurantius

Taxon classificationFungiBoletalesBoletaceae

Ming Zhang & T.H. Li, Phytotaxa 327 (1): 49, 2017

C07D2934-170F-51E0-A1F8-17497AF48080

821522

##### Known distribution.

Central (Hunan Province) and eastern China (Zhejiang Province) (Zhang et al. 2017).

##### Holotype.

GDGM44776 (China, Hunan Province) (non vidi).

##### Notes.

*Chalciporus citrinoaurantius* was originally described from Hunan Province of central China (Zhang et al. 2017); illustrations and a full description of the species have been provided by Zhang et al. (2017).

#### ﻿ Chalciporus hainanensis

Taxon classificationFungiBoletalesBoletaceae

Ming Zhang & T.H. Li, Phytotaxa 327 (1): 47–56, 2017

1D53C5A7-F533-5F4B-BA1A-40BEFD6DAA94

821521

Figs 2i, 6

##### Description.

***Basidiomata*** very small-sized. ***Pileus*** 1–2 cm in diameter, subhemispherical to convex when young, plano-convex to applanate when mature; surface dry, tomentose, light orange-brown (5A3–5A4); context 0.2–0.5 cm in thickness in the center of the pileus, white (1A1), changing red when bruised. ***Hymenophore*** poroid, slightly decurrent; pores angular, about 0.1 cm wide, reddish orange (6A5–6A6); tubes about 0.2 cm long, reddish orange (6A5). ***Stipe*** 1.1–2.3 × 0.2 cm, central, solid, subcylindrical; surface orange-brown to brown (5B4–5B6), but reddish at the apex, with distinctly longitudinal striations; context white (1A1), changing red when bruised; basal mycelium white. ***Odor*** indistinct. ***Taste*** mild.

**Figure 6.**
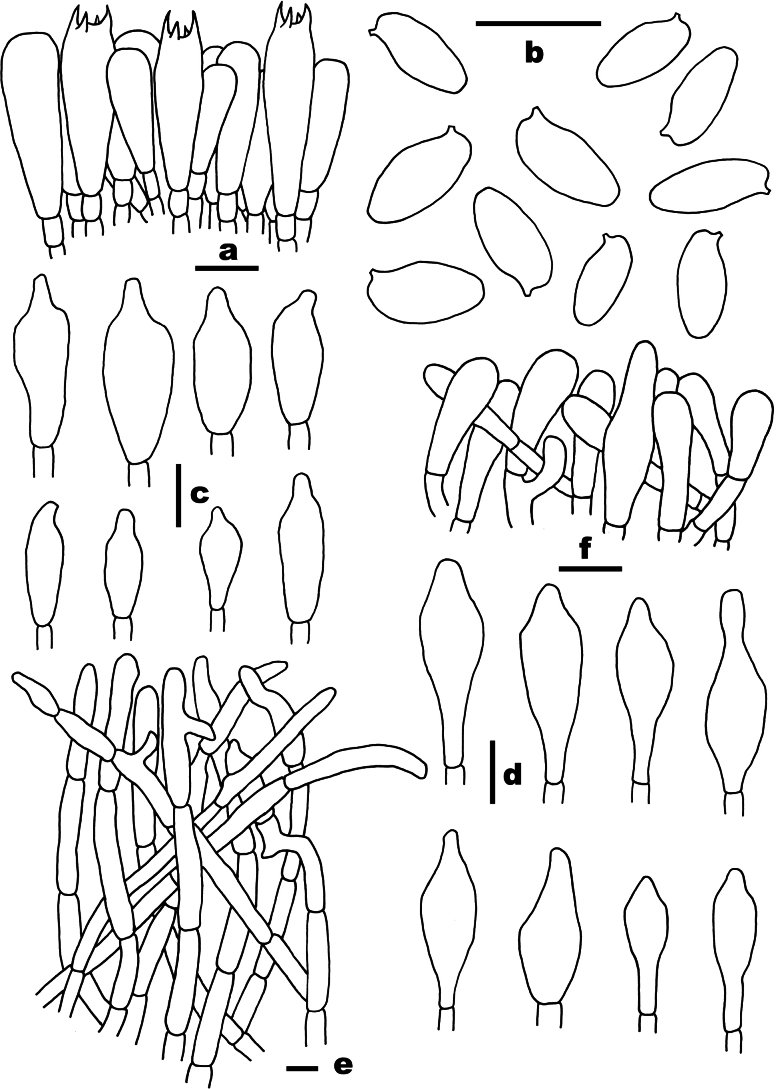
Microscopic features of *Chalciporus hainanensis* (FHMU9951). a. Basidia; b. Basidiospores; c. Cheilocystidia; d. Pleurocystidia; e. Pileipellis; f. Stipitipellis. Scale bars: 10 µm. Drawings by X.N. Li.

***Basidiospores*** [80/4/1] 7.5–10 × 3.5–5 μm, Q= (1.70)1.88–2.50(2.57), Q_m_ = 2.16 ± 0.19, yellowish brown in KOH, elongated to cylindrical, thin-walled, smooth under the light microscope. ***Basidia*** 17–30 × 8–10.5 μm, clavate, thin-walled, 4-spored, colorless in KOH; sterigmata 3–5 μm in length. ***Cheilocystidia*** 17–31 × 6–12 μm, fusiform or subfusiform, slightly thick-walled (up to 1 μm), colorless in KOH. ***Pleurocystidia*** 22–35 × 7–10 μm, fusiform or subfusiform, slightly thick-walled (up to 1 μm), colorless in KOH. ***Hymenophoral trama*** boletoid, yellowish in KOH, thin- to slightly thick-walled (up to 0.5 μm), 3–11 μm wide. ***Pileipellis*** a trichoderm 200–350 μm in thickness, composed of pale yellow in KOH, slightly thick-walled (up to 1 μm) hyphae; terminal cells 20–65 × 7–9 μm, clavate to subcylindrical or cystidioid. ***Pileus trama*** made up of hyphae 1–9 μm in diameter, thin-walled, colorless to pale yellow in KOH. ***Stipitipellis*** a trichoderm-like structure 150–250 μm thick, composed of hyaline to pale yellow in KOH, slightly thin-walled (up to 1 μm) hyphae; terminal cells 10–30× 5–8 μm, clavate or subcylindrical, occasionally fusiform. ***Stipe trama*** composed of parallel hyphae 4–15 μm in diameter, cylindrical, thin-walled, yellow in KOH. ***Clamp connections*** absent in all tissues.

##### Habitat.

Solitary or gregarious on the ground in forests dominated by fagaceous trees(*Castanopsis* spp., *Cyclobalanopsis* spp., *Lithocarpus* spp.).

##### Known distribution.

Southern (Hainan Province) and southeastern China (Fujian Province).

##### Holotype.

GDGM44464 (China, Hainan Province) (non vidi).

##### Materials examined.

China • Fujian Province, Jiangle County, Longqishan National Nature Reserve, elev. 750 m alt. 650 m, 26.4°N, 117.2°E, 21 August 2023, N.K. Zeng8211 (FHMU9951); • Hainan Province, Baisha County, Yinggeling of Hainan Tropical Rainforest National Park, elev. 550 m, 26 June 2015, S. Jiang81 (FHMU4573).

##### Notes.

*Chalciporus hainanensis* was originally described from Hainan Province of southern China (Zhang et al. 2017). In the present study, it was also found to be distributed in Fujian Province of southeastern China. The species is characterized by a very small basidioma, a tomentose, light orange-brown pileus, a reddish-orange hymenophore surface, and a trichodermal pileipellis.

#### ﻿ Chalciporus piperatus

Taxon classificationFungiBoletalesBoletaceae

(Bull.) Bataille, Bull. Soc. Hist. Nat. Doubs 15: 39, 1908

B88FE090-702B-5719-8A07-E4F95672E09D

311021

Boletus piperatus Bull., Herb. Fr. 10 (109–120): t. 451:2, 1790.
Suillus piperatus (Bull.) Poir., Rev. gén. pl.: 498, 1806.
Leccinum piperatum (Bull.) Gray, ANat. Arr. Brit. Pl. 1: 647, 1821.
Viscipellis piperata (Bull.) Quél., Enchir. Fung. Eur. Media Gallia Vig.: 157, 1886.
Ixocomus piperatus (Bull.) Quél., Fl. mycol. Fr.: 414, 1888.
Ceriomyces piperatus (Bull.) Murrill, Mycologia 1 (4): 150, 1909.

##### Known distribution.

Northern China (Shanxi Province), Europe, and North America (Halling et al. 2004; Wu et al. 2014; Xu et al. 2021; Mao et al. 2023); probably southwestern China (Yunnan Province) (Fig. 1).

##### Notes.

*Chalciporus piperatus* was originally described from Europe (Bataille 1908). It was also reported from Shanxi Province, northern China (Mao et al. 2023). Illustrations and a full description of the species have been provided by Moser (1983), Alessio (1985), Muñoz (2005), Šutara et al. (2009), and Mao et al. (2023). It should be noted that *C. pseudopiperatus*, described from Europe, has been considered morphologically close to *C. piperatus* and remains poorly separated based on macroscopic features (Klofac and Krisai-Greilhuber 2020). Although molecular data for *C. pseudopiperatus* exist, its phylogenetic position and taxonomic status have not been fully resolved, and it has been suggested that the species may be more widespread in Europe. Future studies including type and additional European materials will be essential to clarify the relationship between *C. piperatus* and *C. pseudopiperatus*.

#### ﻿ Chalciporus radiatus

Taxon classificationFungiBoletalesBoletaceae

Ming Zhang & T.H. Li, Mycoscience 57 (1): 21, 2015

30A298ED-2A3E-5681-9C7A-69243D36D1F7

811750

##### Known distribution.

Central (Hunan Province), eastern (Zhejiang Province), southeastern (Fujian Province), and southern China (Guangdong and Hainan Provinces) (Zhang et al. 2016; Chai et al. 2019).

##### Holotype.

GDGM 43285 (China, Hunan Province) (non vidi).

##### Materials examined.

China • Zhejiang Province, Qingyuan County, Zuoxi Town, elev. 600 m, 21 August 2024, N.K. Zeng10292 (FHMU8509); • same location and date, N.K. Zeng10296 (FHMU8989).

##### Notes.

*Chalciporus radiatus* was originally described from Hunan Province of central China (Zhang et al. 2016), the Zhejiang specimens cited above extend the range of distribution. Illustrations and a full description of the species have been provided by Zhang et al. (2016) and Chai et al. (2019).

#### ﻿ Chalciporus roseus

Taxon classificationFungiBoletalesBoletaceae

N.K. Zeng & X. Zhang sp. nov.

7BE7A353-7E17-584B-9F89-C9B24EF14E2B

860041

Figs 2j–l, 7

##### Etymology.

Latin “ *roseus*”, referring to pinkinsh to dark reddish pileus.

##### Holotype.

China • Hainan Province, Wuzhishan County, Wuzhishan of Hainan Tropical Rainforest National Park, alt. 600 m, 18.9°N, 109.5°E, 8 May 2024, N.K. Zeng8516 (FHMU7888).

**Figure 7.**
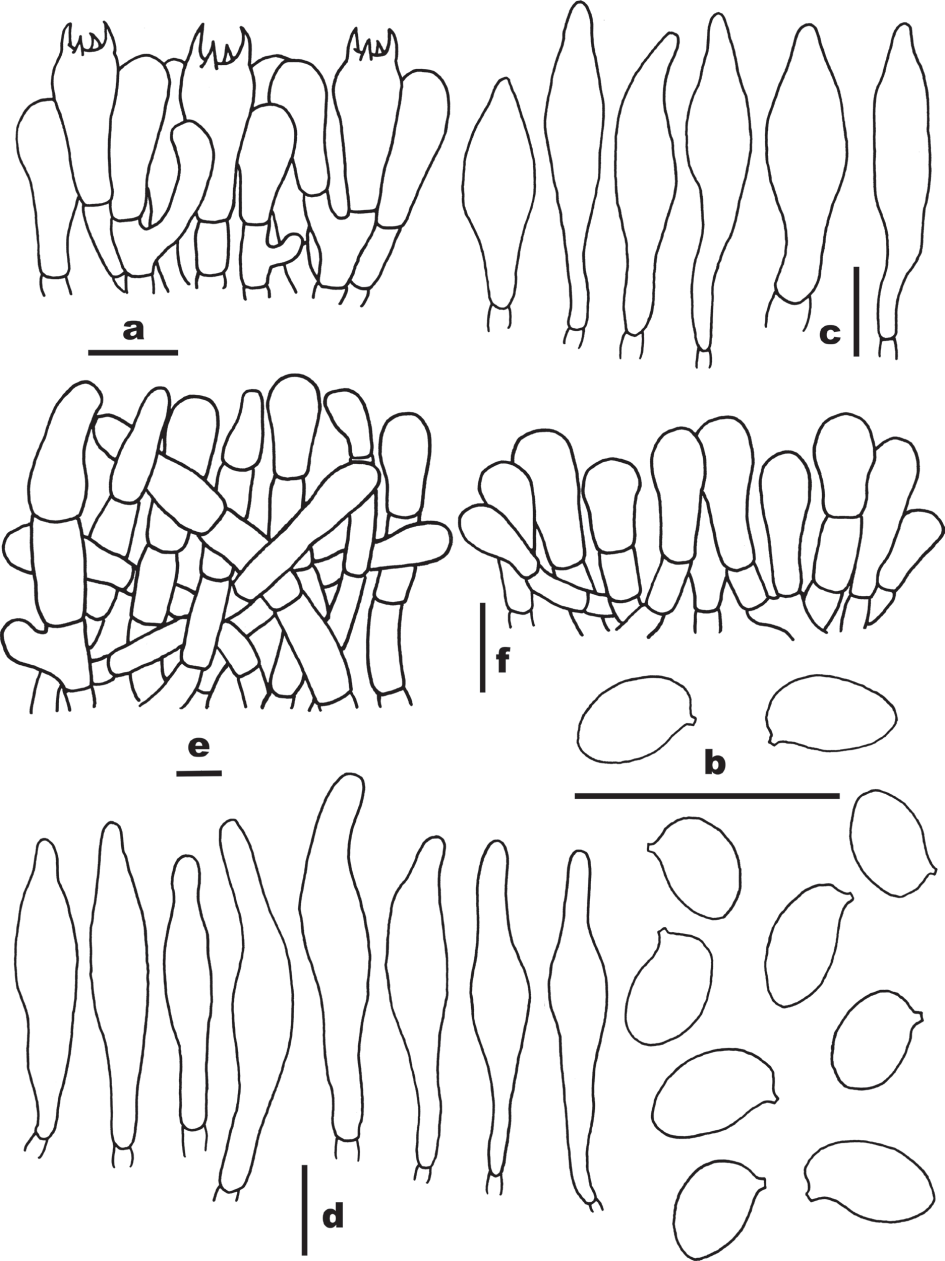
Microscopic features of *Chalciporus roseus* (FHMU7888, holotype). a. Basidia; b. Basidiospores; c. Cheilocystidia; d. Pleurocystidia; e. Pileipellis; f Stipitipellis. Scale bars: 10 µm. Drawings by X.N. Li.

##### Diagnosis.

Differs from closest species of *Chalciporus* by a pinkish to dark reddish pileus with squamules, a yellow hymenophore bruising blue, a pale yellow context changing blue when bruised, broadly ellipsoid to elongate basidiospores measuring 4–5.5 × 3–3.5 μm, and a trichodermal pileipellis.

##### Description.

***Basidiomata*** small-sized. ***Pileus*** 2.8–4.1 cm in diameter, hemispherical to applanate; surface dry, pinkish to dark reddish (11A4–11B5), covered with squamules; context 0.3–0.7 cm in thickness in the center of the pileus, pale yellow (1A2), changing blue when bruised. ***Hymenophore*** poroid, slightly decurrent; pores subangular to roundish, 0.1–0.25 cm wide, yellow (1A4), changing blue in color when bruised; tubes yellowish, changing blue when bruised. ***Stipe*** 4.7–6.2 × 0.4–0.8 cm, central, solid, subcylindrical; surface pinkish to dark reddish (11A4–11B5), with distinctly longitudinal striations; context pale yellow (1A2), changing red when bruised; basal mycelium white. ***Odor*** indistinct. ***Taste*** mild.

***Basidiospores*** [60/3/3] (3.5)4–5.5(6) × (2.5)3–3.5(4) μm, Q= (1.14)1.17–1.83(2.0), Q_m_ = 1.54 ± 0.18, yellowish brown in KOH, broadly ellipsoid, ellipsoid to elongate, slightly thick-walled (0.8–1 µm), smooth under the light microscope. ***Basidia*** 7–22 × 4–8 μm, clavate, thin-walled, 4-spored, colorless to pale yellow in KOH; sterigmata 2–6 μm in length. ***Cheilocystidia*** 26–44 × 5–9 μm, fusiform or subfusiform, slightly thick-walled (up to 1 μm), colorless to pale yellow in KOH. ***Pleurocystidia*** 19–43 × 5–8 μm, fusiform or subfusiform, thin-walled, colorless to pale yellow in KOH. ***Hymenophoral trama*** boletoid, colorless to yellowish in KOH, thin- to slightly thick-walled (up to 0.5 μm), 9–15 μm wide. ***Pileipellis*** a trichoderm 150–250 μm in thickness, composed of colorless to pale yellow in KOH, slightly thick-walled (up to 1 μm) hyphae; terminal cells 14–40 × 6–10 μm, clavate to subcylindrical or cystidioid. ***Pileus trama*** made up of hyphae 5–19 μm in diameter, slightly thick-walled (up to 1 μm), colorless in KOH. ***Stipitipellis*** a trichoderm-like structure 700–800 μm thick, composed of hyaline to pale yellow in KOH, thin-walled hyphae; terminal cells 10–29× 4.5–9 μm, clavate or subcylindrical. ***Stipe trama*** composed of parallel hyphae 3–7 μm diameter, cylindrical, thin-walled, yellow in KOH. ***Clamp connections*** absent in all tissues.

##### Habitat.

Solitary or scattered on the ground in forests dominated by fagaceous trees (*Lithocarpus* spp.).

##### Known distribution.

Southern China (Hainan Province).

##### Additional materials examined.

China • Hainan Province: Wuzhishan County, Wuzhishan of Hainan Tropical Rainforest National Park, alt. 600 m, 18.9°N, 109.5°E, 8 May 2024, N.K. Zeng8516-1 (FHMU11614); • same location and date, N.K. Zeng8516-2 (FHMU11615).

##### Notes.

Phylogenetically, *C. roseus* is closely related to lineage 5, which includes two collections from southwestern China (Fig. 1). However, since both specimens representing this lineage are immature, detailed morphological comparisons will be performed in future studies when mature specimens become available. Morphologically, the feature of the dark reddish pileus and yellow hymenophoral surface of *C. roseus* distinguishes it from other species in the genus *Chalciporus*.

#### ﻿ Chalciporus rubinelloides

Taxon classificationFungiBoletalesBoletaceae

G.Wu & Zhu L. Yang, Fungal Diversity 81: 74, 2016

241BE551-5D90-573D-B164-0F3DB39582E1

818400

##### Known distribution.

Southwestern China (Yunnan Province) (Wu et al. 2016).

##### Holotype.

HKAS74952 (China, Yunnan Province) (non vidi).

##### Notes.

*Chalciporus rubinelloides* was originally described from Yunnan Province of southwestern China (Wu et al. 2016); illustrations and a full description of the species have been provided by Wu et al. (2016).

#### ﻿ Chalciporus sinensis

Taxon classificationFungiBoletalesBoletaceae

N.K. Zeng, Chang Xu, S. Jiang & Zhi Q. Liang, Mycol. Progr. 20 (12): 1576, 2021

B3E9DD7E-B92B-5AB7-96D5-072C7E371A4B

839317

##### Known distribution.

Southern China (Hainan Province) (Xu et al. 2021).

##### Holotype.

N.K. Zeng4478 (FHMU4701) (China, Hainan Province) (vidi).

##### Notes.

*Chalciporus sinensis* was originally described from Hainan Province of southern China (Xu et al. 2021); illustrations and a full description of the species have been provided by Xu et al. (Xu et al. 2021).

#### ﻿ Chalciporus vulparius

Taxon classificationFungiBoletalesBoletaceae

N.K. Zeng, Chang Xu & Zhi Q. Liang, Mycol. Progr. 20 (12): 1578, 2021

B618092E-B770-59C3-9E8C-61FFA59FDBD5

839318

##### Known distribution.

Southern China (Hainan Province) (Xu et al. 2021).

##### Holotype.

N.K. Zeng4979 (FHMU5560) (China, Hainan Province) (vidi).

##### Notes.

*Chalciporus vulparius* was originally described from Hainan Province of southern China (Xu et al. 2021); illustrations and a full description of the species have been provided by Xu et al. (Xu et al. 2021).

### ﻿Key to accepted *Chalciporus* species in China

**Table:** 

1	Hymenophoral surface yellow	**2**
–	Hymenophoral surface reddish-orange to reddish	**3**
2	Pileal surface pale brown to brown, tomentose, context unchanging in color when bruised	***C. brunneus***
–	Pileal surface pinkish to dark reddish, covered with squamules, context changing blue when bruised	***C. roseus***
3	Pores arranged radially when young (appearing sublamellate at maturity)	***C. radiatus***
–	Pores not arranged radially	**4**
4	Pileal context changing pink or red when bruised	**5**
–	Pileal context unchanging in color when bruised	**6**
5	Basidiospores 9–11 × 3–3.5 μm	***C. piperatus***
–	Basidiospores 7.5–10 × 3.5–5 μm	***C. hainanensis***
6	Pileal surface distinctly squamulose (with scales or squamules)	**7**
–	Pileal surface nearly glabrous, velvety-tomentose, or tomentose, without distinct scales or squamules	**8**
7	Pileal surface covered with brown to grey-brown scales	***C. sinensis***
–	Pileal surface covered with orange to reddish squamules	***C. aurantiolepidotus***
8	Basidiomata small to medium (pileus up to 7 cm in diameter), basidiospores longer (up to16 μm long)	***C. rubinelloides***
–	Basidiomata very small to small (pileus up to 5 cm in diameter), basidiospores shorter (up to 13 μm long)	**9**
9	Pileal surface reddish brown to pale reddish brown, basidiospores shorter measuring 5.5–9 × 3–5 μm	***C. vulparius***
–	Pileal surface light yellow to greyish orange, basidiospores longer measuring 9.5–12.5 × 3.5–4 μm	***C. citrinoaurantius***

#### ﻿ Pseudophylloporus

Taxon classificationFungiBoletalesBoletaceae

N.K. Zeng, H.Z. Qin, W.F. Lin & L.G. Hu, J. Fungi 10: 7. 2024

8D15743A-AAE0-5D96-B487-280431DC9695

Pseudophylloporus , typified by P. baishanzuensis, was initially established to accommodate species characterized by a lamellate hymenophore usually with forked lamellae (Qin et al. 2024). Until recently, the genus contained only a single known species, viz. P. baishanzuensis, which was uncovered in subtropical evergreen broad-leaved forests in eastern China. In the present study, a second species of Pseudophylloporus is described based on morphological features and phylogenetic evidence.

#### ﻿ Pseudophylloporus baishanzuensis

Taxon classificationFungiBoletalesBoletaceae

N.K. Zeng, H.Z. Qin, W.F. Lin & L.G. Hu, J. Fungi 10: 8, 2024

62CFFCC3-2EDD-55EC-BC25-3802B33EBB24

855764

##### Known distribution.

Eastern China (Zhejiang Province) (Qin et al. 2024).

##### Holotype.

N.K. Zeng7702 (FHMU7694) (China, Zhejiang Province) (vidi).

##### Notes.

*Pseudophylloporus baishanzuensis* was originally described from Zhejiang Province of eastern China (Qin et al. 2024); illustrations and a full description of the species have been provided by Qin et al. (2024).

#### ﻿ Pseudophylloporus castaneus

Taxon classificationFungiBoletalesBoletaceae

N.K. Zeng, X. Zhang & J.B. Pu sp. nov.

0A53BB28-1E14-515F-B8A9-E86CB7E92D13

860042

Figs 2m–o, 8

##### Etymology.

Latin “*castaneus*”, referring to the chestnut-brown pileus.

**Figure 8.**
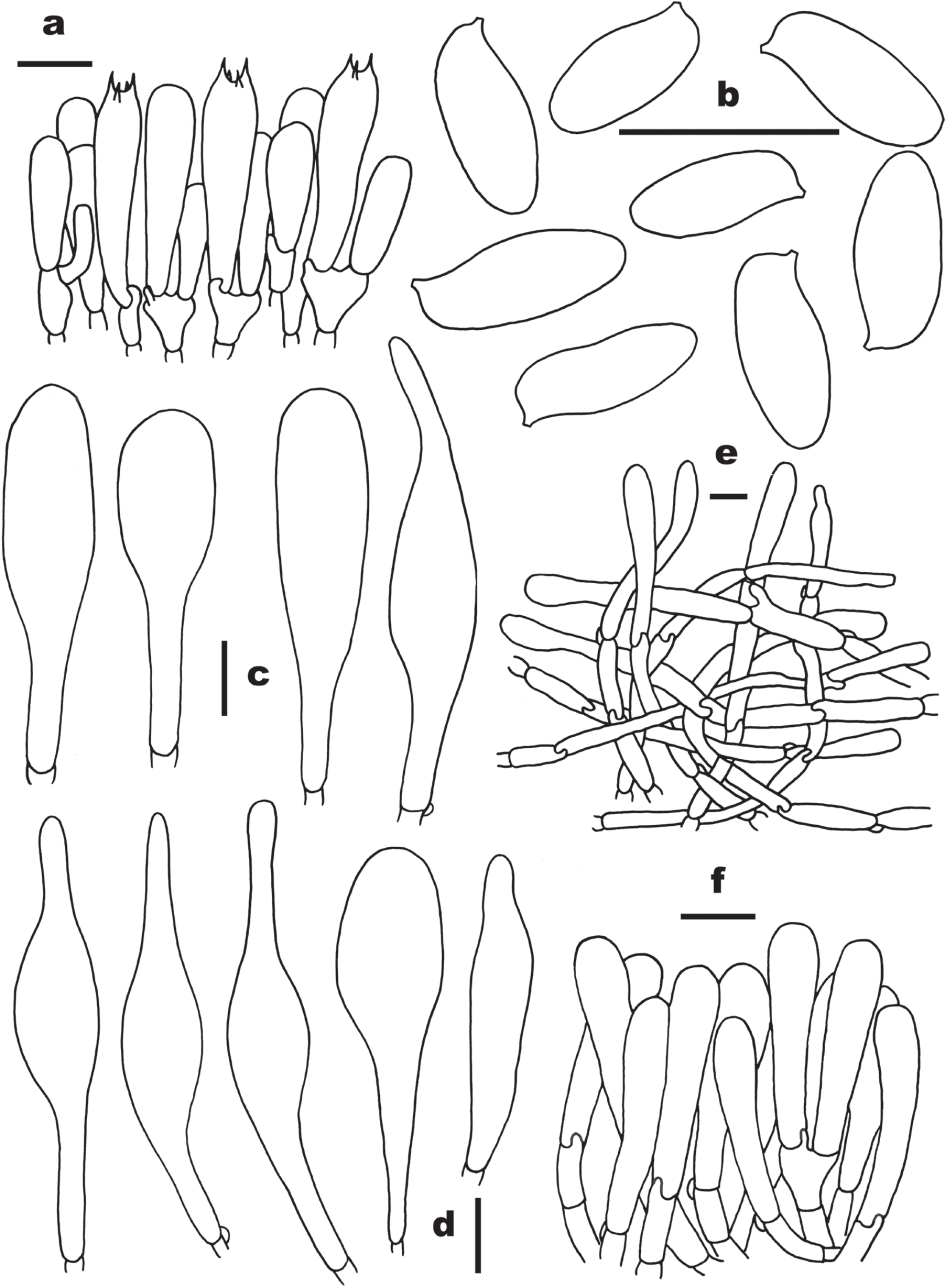
Microscopic features of *Pseudophylloporus castaneus* (ZJMR330727230331, holotype). a. Basidia; b. Basidiospores; c. Cheilocystidia; d. Pleurocystidia; e. Pileipellis; f. Stipitipellis. Scale bars: 10 µm. Drawings by X.N. Li.

##### Holotype.

China • Zhejiang Province, Pan’an County, Dapanshan National Nature Reserve, alt. 1003 m, 29.0°N, 120.5°E, 7 July 2023, FC330727230331 (ZJMR330727230331).

##### Diagnosis.

Differs from closest species of *Pseudophylloporus* by a chestnut-brown pileus, cystidia with golden or yellowish-brown plasmatic pigments, and an intricate trichodermal pileipellis.

##### Description.

***Basidiomata*** very small-sized. ***Pileus*** 2.5–3 cm in diameter, subhemispherical when young, then subhemispherical to plano-convex; surface dry, tomentose, chestnut-brown (5E7–5E8); context 0.3–0.6 cm in thickness in the center of the pileus, white (1A1), changing bluish when injured. ***Hymenophore*** lamellate, free; lamellae 0.1–0.3 cm in height, subdistant, usually forked, brown (5D4), turning blue quickly. ***Stipe*** 1.7–2.6 × 0.3–0.5 cm, clavate or tapering upwards, solid; surface yellow (4A3) to yellowish-brown (4C5), with purple-reddish (10E3) longitudinal striations; context pale yellow (2A2), changing blue slightly when injured; basal mycelium yellowish (2A4). ***Odor*** indistinct. ***Taste*** mild.

***Basidiospores*** [60/3/3] 8–9.5(10) × 3–4(4.5) μm, Q = 2.0–3.0, Q_m_ = 2.44 ± 0.29, yellowish brown in KOH, fusoid to cylindrical, slightly thick-walled (0.8–1 µm), smooth under the light microscope. ***Basidia*** 24–34 × 5–8 μm, clavate, slightly thick-walled (0.8–1 µm), 4-spored, colorless to pale yellow in KOH; sterigmata 2–5 μm in length. ***Cheilocystidia*** 43–65 × 10–15 μm, abundant, ventricose, subclavate or subfusiform, slightly thick-walled (up to 1 μm), with golden or yellowish brown plasmatic pigment in KOH. ***Pleurocystidia*** 41–65 × 8–15 μm, subclavate or subfusiform, thin- to slightly thick-walled (up to 1 μm), with golden or yellowish brown plasmatic pigment in KOH. ***Hymenophoral trama*** boletoid, colorless to yellowish in KOH, thin- to slightly thick-walled (up to 0.5 μm), 4–17 μm wide. ***Pileipellis*** an intricate trichoderm 150–350 μm in thickness, composed of light yellow in KOH, slightly thick-walled (up to 1 μm) hyphae; terminal cells 25–95 × 4–9 μm, clavate to subcylindrical or cystidioid. ***Pileus trama*** made up of hyphae 4–10 μm in diameter, slightly thick-walled (up to 1 μm), yellow in KOH. ***Stipitipellis*** a trichoderm-like structure 30–100 μm thick, composed of pale yellow in KOH, slightly thick-walled (up to 1 μm) hyphae, terminal cells 30–60 × 5–6 μm, subclavate or clavate. ***Stipe trama*** composed of parallel hyphae 5–30 μm in diameter, cylindrical, thin- to slightly thick-walled (up to 0.5 μm), pale yellow in KOH. ***Clamp connections*** present in all tissues.

##### Habitat.

Solitary or scattered on the ground in forests dominated by fagaceous trees (*Quercus* spp.)

##### Known distribution.

Eastern China (Zhejiang Province).

##### Additional materials examined.

China • Zhejiang Province, Pan’an County, Dapanshan National Nature Reserve, alt. 1003 m, 28.9°N, 120.5°E, 28 June 2023, FC330727230331-1 (FHMU11549); • same location and date, FC330727230331-2 (FHMU11550).

##### Notes.

*Pseudophylloporus castaneus* is phylogenetically related and morphologically similar to *P. baishanzuensis*. However, *P. baishanzuensis* has a yellowish-brown to pale brown pileus, a stipe densely covered with pale brown scales, cystidia without golden or yellowish brown plasmatic pigments, and a cutis-type pileipellis (Qin et al. 2024).

### ﻿Key to accepted *Pseudophylloporus* species in China

**Table:** 

1	Pileal suface chestnut-brown, stipe without pale brown scales, cystidia with golden or yellowish-brown plasmatic pigment, pileipellis trichodermal	***P. castaneus***
–	Pileal suface yellowish-brown to pale brown, stipe densely covered with pale brown scales, cystidia without golden or yellowish brown plasmatic pigment, pileipellis a cutis	***P. baishanzuensis***

## ﻿Discussion

This study clearly reveals the species diversity and taxonomic composition of the subfamily Chalciporoideae in China. A total of 16 phylogenetic species-level lineages were identified, including 2 in *Buchwaldoboletus*, 12 in *Chalciporus*, and 2 in *Pseudophylloporus*. Further detailed morphological observations show four new species are described herein (3 in *Chalciporus* and 1 in *Pseudophylloporus*), and 10 known species are confirmed to occur in China (2 in *Buchwaldoboletus*, 7 in *Chalciporus*, and 1 in *Pseudophylloporus*) (Table 2). These results not only contribute to our pertinent knowledge of the Chalciporoideae subfamily but also provide a foundation for further studies on the subfamily’s biogeography and ecological adaptation.

**Table 2.** List of described, reported or controversial Chalciporoideae species in China.

Species	Type locality	Treatment	References
*Buchwaldoboletus lignicola*	Germany	Accepted	Pilát (1969); Wu et al. (2016)
*Buchwaldoboletus xylophilus*	Sri Lanka	Accepted	Ortiz-Santana and Both (2011); Xie et al. (2021)
*Chalciporus aurantiolepidotus*	Hainan, southern China	Accepted	This study
*Chalciporus brunneus*	Hainan, southern China	Accepted	This study
*Chalciporus citrinoaurantius*	Hunan, central China	Accepted	Zhang et al. (2017)
*Chalciporus hainanensis*	Hainan, southern China	Accepted	Zhang et al. (2017)
*Chalciporus piperatus*	Europe	Accepted	Bataille (1908); Mao et al. (2023)
*Chalciporus radiatus*	Hunan, central Chin	Accepted	Zhang et al. (2016); Zhang et al. (2017)
*Chalciporus roseus*	Hainan, southern China	Accepted	This study
*Chalciporus rubinelloides*	Yunnan, SW China	Accepted	Wu et al. (2016)
*Chalciporus sinensis*	Hainan, southern China	Accepted	Xu et al. (2021)
*Chalciporus vulparius*	Hainan, southern China	Accepted	Xu et al. (2021)
*Rubinoboletus ballouii*	Singapore	Transferred to *Tylopilus*	Singer (1947)
Rubinoboletus ballouii var. fuscatus	Singapore	Transferred to *Tylopilus*	Heinemann and Rammeloo (1983); Li and Yang (2021)
*Pseudophylloporus baishanzuensis*	Zhejiang, eastern China	Accepted	Qin et al. (2024)
*Pseudophylloporus castaneus*	Zhejiang, eastern China	Accepted	This study

Chalciporoideae represents one of the earliest diverging lineages within Boletaceae, yet its classification has long been controversial. Recent phylogenetic studies have confirmed that *Chalciporus*, *Buchwaldoboletus*, and the newly defined genus *Pseudophylloporus* constitute a well-supported monophyletic group (Qin et al. 2024; Tremble et al. 2024). However, *Rubinoboletus* Pilát & Dermek was originally described as a monotypic genus, and its type species, *R. rubinus* (W.G. Sm.) Pilát & Dermek, has been conclusively shown to nest within *Chalciporus*, making *Rubinoboletus* a synonym of the latter. Nevertheless, the placement of other species that were subsequently assigned to *Rubinoboletus* remains unresolved. Based on early morphological studies, *Rubinoboletus* was considered synonymous with *Chalciporus* (Singer 1973; Pegler and Young 1981; Klofac 2006), with Klofac (2006) further proposing its treatment as a subgenus. Recent genome-scale analyses also confirmed that the type species of *Rubinoboletus*, *R. rubinus* (W.G. Sm.) Pilát & Dermek, belongs firmly within the *Chalciporus* clade, thus validating the synonymy of the two genera (Tremble et al. 2024). However, this taxonomic resolution does not extend to all species classified in *Rubinoboletus*. For instance, *R. phaseolisporus* T.H. Li, R.N. Hilton & Watling was reclassified into the genus *Tylopilus* P. Karst. based on a combination of morphological characteristics and multilocus phylogenetic analyses (Osmundson et al. 2021). The reclassification of *Rubinoboletus* highlights its polyphyletic nature and emphasizes the necessity of conducting species-level systematic analyses across the genus. Additionally, the genus *Nevesoporus* A.C. Magnago & T.W. Henkel was originally placed within the subfamily Chalciporoideae at the time of its establishment (Magnago et al. 2022). However, subsequent genome-scale analyses and multilocus phylogenetic studies have confirmed that this genus should be assigned to Boletoideae rather than Chalciporoideae (Osmundson et al. 2021; Qin et al. 2024). These findings underscore the necessity of establishing a comprehensive taxonomic framework that integrates morphological, molecular, and ecological evidence to achieve more accurate phylogenetic placement and taxonomic refinement.

Our study also highlights unexpected morphological diversity within the genus *Chalciporus*. Traditionally, the presence of a distinctly reddish hymenophore has been regarded as one of the key diagnostic features of the genus. However, exceptions are known, such as *C. hypochryseus* described by Šutara (1993), which is characterized by yellow pores, a feature also documented by subsequent authors (Galli 1998; Simonini 1998; Muñoz 2005; Šutara et al. 2009). Consistent with this, our newly described species, *C. brunneus* and *C. roseus*, also exhibit a pale-yellow to yellow hymenophore, differing significantly from the conventional reddish pigmentation. The discovery of these taxa further suggests that the current generic concept of *Chalciporus* may need to be revised or expanded to accommodate a wider spectrum of hymenophore coloration.

Notably, many species in the Boletaceae exhibit pronounced geographic restriction, with truly widespread taxa being relatively rare (Chai et al. 2019; Xie et al. 2021; Xue et al. 2023; Qin et al. 2024). *Chalciporus piperatus*, originally described from Europe, has been widely reported in China based solely on morphological characters (Bi et al. 1993; Li and Song 2002), which has long raised doubts about its actual presence in the region (Xu et al. 2021). Recently, Mao et al. (2023) confirmed its occurrence in northern China (Shanxi Province) using both morphological and molecular phylogenetic evidence. Interestingly, in our phylogenetic analyses, sequences from Chinese specimens clustered with those from Germany, albeit with relatively low statistical support (Fig. 1). Given the geographic distribution pattern of *C. piperatus*, it is plausible that the European species *C. pseudopiperatus*, closely related to *C. piperatus* (Klofac and Krisai-Greilhuber 2020), may also occur in China. Therefore, further sampling and reassessment of specimens currently labelled as “*C. piperatus*” from China are strongly recommended.

Our study further highlights the remarkable diversity of Chalciporoideae in southern China, particularly in subtropical and tropical regions. The majority of species in this subfamily exhibit narrow geographic distributions, and the newly identified taxa are predominantly located in the same climatic zones. This distribution pattern suggests that the subtropical-tropical region of China represents a primary center of species diversity for Chalciporoideae. An exceptional case within our study is *B. lignicola*, the only species found to occur widely across both temperate and tropical regions of China. Its broad ecological amplitude and saprophytic or possibly mycoparasitic lifestyle suggest that it may hold potential for artificial cultivation.

Despite significant advances in recent years, the trophic modes of Chalciporoideae remain poorly understood. Members of this subfamily may adopt a range of nutritional strategies, including ectomycorrhizal, saprotrophic, and mycoparasitic lifestyles. Although some studies have suggested that species such as *C. piperatus* and *B. lignicola* may not be ectomycorrhizal (Dickie and Johnston 2008; Dickie et al. 2010; Robinson 2010), there is still a lack of systematic understanding of the subfamily’s overall ecological traits. To elucidate its trophic spectrum, further integrative ecological studies will be necessary. Such investigations are not only essential for revealing the ecological plasticity within Chalciporoideae but also for enhancing our understanding of the evolutionary dynamics of nutritional modes in early-diverging bolete lineages.

In conclusion, this study provides new insights into the species diversity and phylogenetic relationships of Chalciporoideae in China. The discovery of novel taxa and the confirmation of known species distributions underscore the importance of subtropical and tropical China as a biodiversity hotspot for the subfamily. Continued extensive field surveys, coupled with integrative taxonomic and multidisciplinary approaches, will be vital for fully uncovering the ecological and evolutionary complexity of this important fungal group.

## ﻿Conclusion

Although a number of species within Chalciporoideae have been reported worldwide, the diversity and phylogenetic relationships of this subfamily remain insufficiently understood, especially in China. In the present study, four new species of Chalciporoideae were described, two previously known species were redescribed, and an additional eight known species were reviewed based on morphological characteristics and molecular phylogenetic analyses. Our findings provide new insights into the species composition within Chalciporoideae, significantly enhancing our understanding of the Boletaceae family in China.

## Supplementary Material

XML Treatment for Buchwaldoboletus

XML Treatment for Buchwaldoboletus lignicola

XML Treatment for Buchwaldoboletus xylophilus

XML Treatment for Chalciporus

XML Treatment for Chalciporus aurantiolepidotus

XML Treatment for Chalciporus brunneus

XML Treatment for Chalciporus citrinoaurantius

XML Treatment for Chalciporus hainanensis

XML Treatment for Chalciporus piperatus

XML Treatment for Chalciporus radiatus

XML Treatment for Chalciporus roseus

XML Treatment for Chalciporus rubinelloides

XML Treatment for Chalciporus sinensis

XML Treatment for Chalciporus vulparius

XML Treatment for Pseudophylloporus

XML Treatment for Pseudophylloporus baishanzuensis

XML Treatment for Pseudophylloporus castaneus

## ﻿Additional information

### Use of AI

No use of AI was reported.

### Author ORCIDs

Xu Zhang https://orcid.org/0000-0003-1283-0573

Xin-Ni Li https://orcid.org/0009-0000-8069-5241

Wei-Qing Liang https://orcid.org/0009-0003-2948-0845

Xiao-Dong Mu https://orcid.org/0009-0009-9375-0727

Ye-Fei Yu https://orcid.org/0009-0003-4890-5845

Xiao-Jun Wu https://orcid.org/0009-0001-4184-6331

Si-Yu Chen https://orcid.org/0009-0003-6597-3081

Jin-Bao Pu https://orcid.org/0000-0003-4911-1169

Nian-Kai Zeng https://orcid.org/0000-0001-7651-4524

### Data availability

All of the data that support the findings of this study are available in the main text.
